# Elastic quantum coupling between free electrons and photons

**DOI:** 10.1126/sciadv.aed3278

**Published:** 2026-09-04

**Authors:** Dingguo Zheng, Ofer Kfir

**Affiliations:** School of Electrical and Computer Engineering, The Iby and Aladar Fleischman Faculty of Engineering, Tel Aviv University, Tel Aviv 69978, Israel.

## Abstract

The quantum coupling between free electrons and photons enables the application of quantum optics techniques in electron microscopy. For example, the phase of the electron wave function can be manipulated by passing the electron through an intense laser field. We develop here a quantum-optical framework for elastic coupling, which reveals the hidden back action: the phase shift that a passing electron induces on the photonic mode. The so-called “phase kickback” can be quantified as a refractive index of a free electron or be formulated as a dispersive Hamiltonian. This elastic scattering paves the way toward electron number states by counting free electrons nondestructively, and thus, allowing for subshot-noise sensing and imaging at the angstrom-scale.

## INTRODUCTION

Electron microscopes (EM) play a versatile role in science, enabling, on one hand, imaging and spectroscopy at the ultimate atomic-scale resolution (*1*, *2*) and, on the other hand, acting as a testbed for the physics of fundamental constituents of the standard model—electrons and photons, matter, and light (*3*–*5*). There is an ongoing pursuit to understand and control the quantum light-electron interaction to facilitate unforeseen contrast mechanisms and enhance measurement precision. Studies on laser manipulation of electron beams have explored various ways to structure their wave function (*6*–*10*). Inelastic approaches use lasers to modulate the electron spectral density (*3*–*5*, *11*–*13*), enabling electron bunching into attosecond pulses (*14*–*17*), electron deflection (*17*–*19*), or control of the spatial profile (*20*–*22*). In addition to laser-driven or stimulated processes, spontaneous inelastic electron-photon coupling required a second-quantization treatment (*23*–*28*). The inelastic coupling amplitude, $g_{\text{Qu}}$, was established, and subsequent theoretical and experimental studies were able to push it toward unity, that is, one photon generation for each electron on average (*29*–*31*). The detection of photons emitted by a passing electron enables the creation of heralded and entangled electrons within EM (*32*–*36*), forming nonclassical states that may be used to boost performance. However, inelastic interactions are destructive because the final electron state is distinguishable from its initial state. In holographic experiments, for example, if one segment of an electron wave function undergoes such inelastic coupling, then it loses its coherence and suppresses the hologram’s visibility.

For elastic coupling, there is ample theory and experiments on stimulated interactions, based on the phase that an intense laser field induces on the electron wave function. The most famous example is the Kapitza-Dirac effect (*37*–*39*), where standing optical lattices result in discrete electron diffraction peaks. Today, lasers can apply an electron-phase retarder for holographic imaging (*40*–*43*), and dress the electron wave function arbitrarily (*44*, *45*), or use it for specific use cases as aberration correction (*46*–*48*). However, if one asks what effects the electron has on the intense optical field, then there is no outstanding means to address that.

Here, we develop a quantum-optical framework for elastic electron-photon coupling, which encompasses both laser-induced and spontaneous phenomena. Using second-quantization formalism for both the electrons and the photons, we show where the laser-based electron-phase retardation arises from and reveal its fundamental back action on the photon field. The electron contributes an additional index of refraction for the light field, which is birefringent. The dispersive effect is exemplified for various quantum-optical states, such as coherent, squeezed, Fock, and N00N states. Furthermore, we propose a quantum nondemolition (QND) measurement of the electron number in a beam based on this free-electron–induced optical dispersion, akin to QND measurements of the photon number (*49*–*52*). Ultimately, harnessing this elastic quantum scattering could enable the generation of sub-Poissonian electron statistics for quantum sensing and imaging.

## RESULTS

### Quantum theory of elastic interaction

This section briefly describes the reduction of the electron-photon coupling Hamiltonian into a form of phase-only, QND scattering operator, on which the effects in this manuscript rely. We begin by following the derivations of García de Abajo and colleagues (*6*, *44*, *53*), considering a propagating spinless electron along the *z* axis with an initial energy $\varepsilon_{0}$ and momentum $p_{0}=p_{0}\hat{z}$. The electron’s relativistic parameters are $\beta =v_{0}/c$ and $\gamma =1/\sqrt{1-\beta^{2}}$, where c is the speed of light and $v_{0}=|v_{0}|$ is the electron speed. The vector potential operator of monochromatic light fields can be expressed as $\hat{A}=A\hat{a}e^{-i\omega t}+A^{*}{\hat{a}}^{†}e^{i\omega t}$, where $A=-\frac{i}{\omega }E\left(r\right)$. ω is the optical angular frequency, $\hat{a}$ (${\hat{a}}^{†}$) is photon annihilation (creation) operator for a particular optical mode, and E(r) is the electric field in position r associated with a single photon in that mode. Electron states are described by ${\hat{c}}_{k}$ and ${\hat{c}}_{k}^{†}$, the fermionic annihilation and creation operators, respectively, for an electron with wave vector $k\hat{z}$. An initial state with $N_{e}$ electrons can be written as $|\psi_{e}\rangle =\prod_{k=k_{1}}^{k_{N_{e}}}{\hat{c}}_{k}^{†}{|0\rangle }_{e}$, where ${|0\rangle }_{e}$ is the electron vacuum state. In the rest of this article, $\Pi_{k=k_{1}}^{k_{N_{e}}}$ is simply marked as $\Pi_{k}$ for brevity. The initial state of the system can be written as a joint electron-photon state, $|\Psi_{i}\rangle =|\psi_{\text{ph}}\rangle ⊗|\psi_{e}\rangle$.

The system’s Hamiltonian, $\hat{H}={\hat{H}}_{0}+{\hat{H}}_{\text{int}}$, includes the uncoupled term and interaction term. The latter is derived explicitly in text S1. However, a short outline of the derivation appears here to point to the origin of the elastic electron-photon coupling. We expand the Hamiltonian to a second order around an electron energy $\varepsilon_{0}$ and use the minimal coupling scheme with the mechanical momentum $\hat{p}+e\hat{A}$, where e is the elementary charge and $\hat{p}$ is the canonical momentum. ${\hat{H}}_{0}$ sums the independent electron and photon terms, ${\hat{H}}_{0}={\hat{H}}_{\text{ph}}+{\hat{H}}_{e}$, where

Hˆph=ℏω(aˆ†aˆ+12), Hˆe=∑k=k1kNeεk,0cˆk†cˆk (1)

For simplicity, we consider nearly monochromatic electrons, $\varepsilon_{k,0}\approx \varepsilon_{0}$. In the rest of this article, $\Sigma_{k=k_{1}}^{k_{N_{e}}}$ is simply marked as $\Sigma_{k}$. ${\hat{H}}_{\text{int}}$ can be expressed as ${\hat{H}}_{\text{int}}=\sum_{k}\left({\hat{H}}_{1}+{\hat{H}}_{2}+{\hat{H}}_{\phi }\right)\;{\hat{c}}_{k}^{†}{\hat{c}}_{k}$. We separate it into two terms related to phase matching, ${\hat{H}}_{1}=ev_{0}\cdot A\hat{a}e^{-i\omega t}+h.c.$, ${\hat{H}}_{2}=\frac{e^{2}}{2\gamma m_{e}}A^{T}\Gamma A{\hat{a}}^{2}e^{-2i\omega t}+h.c.$, where h.c. is the Hermitian conjugate. The third is an elastic, real-valued term, independent of phase-matching

$${\hat{H}}_{\phi }=\frac{e^{2}}{\gamma m_{e}}A^{†}\Gamma A\left({\hat{a}}^{†}\hat{a}+\frac{1}{2}\right)$$ (2)

$m_{e}$ is the electron rest mass. Γ is a 3 × 3 matrix, called here the birefringence matrix for reasons we discuss later. For electrons traveling along the *z* direction, Γ reduces to $\text{diag}\left(1,1,\frac{1}{\gamma^{2}}\right)$.

In the interaction picture, the evolution is governed by the scattering operator, $\hat{S}$, by $|\Psi_{f}\rangle =\hat{S}|\Psi_{i}\rangle$. By solving the Schrödinger equation, we obtain the scattering operator

$$\hat{S}=e^{i\sum_{k}{\hat{c}}_{k}^{†}{\hat{c}}_{k}\chi }e^{\sum_{k}{\hat{c}}_{k}^{†}{\hat{c}}_{k}\left(g_{\text{Qu}}\hat{b}{\hat{a}}^{†}-g_{\text{Qu}}^{*}{\hat{b}}^{†}\hat{a}+\frac{1}{2}g_{2}^{*}{\hat{b}}^{†2}{\hat{a}}^{2}-\frac{1}{2}g_{2}{\hat{b}}^{2}{\hat{a}}^{†2}-ig_{\phi }\left({\hat{a}}^{†}\hat{a}+\frac{1}{2}\right)\right)}$$ (3)

We refer to text S2 for a detailed derivation. Here, $\hat{b}$ and ${\hat{b}}^{†}$ are the commuting electron-energy–ladder operators (*23*, *24*) in the nonrecoil approximation, and $g_{\text{Qu}}$ (*23*–*25*, *32*, *33*) and $g_{2}$ are the first- and second-order inelastic quantum coupling amplitudes emanating from ${\hat{H}}_{1}$ and ${\hat{H}}_{2}$, respectively. The final term is the elastic coupling, with the coefficient

$$g_{\phi }=\frac{e^{2}}{\hbar p_{0}}\int_{-\infty }^{+\infty }\mathrm{dz}A^{†}\Gamma A$$ (4)

which originates as an integral over ${\hat{H}}_{\phi }$ along electron trajectory. $g_{\phi }$ is a real number because the Hermitian quadratic integrand $A^{†}\Gamma A$ is real. Thus, $g_{\phi }$ contributes pure phase effects to electron-photon scattering. This is the elastic coupling term we emphasize in this work. The additional phase term, χ, was investigated in detail in (*54*) for single electrons. It originates from the noncommuting nature of the interaction Hamiltonian at different times, $\left[{\hat{H}}_{\text{int}}\left(t_{1}\right),{\hat{H}}_{\text{int}}\left(t_{2}\right)\right]\neq 0$. To the leading order of $\left[{\hat{H}}_{1}\left(t_{1}\right),{\hat{H}}_{1}\left(t_{2}\right)\right]$, one finds that

$$\chi =-\text{Im}\left\{\int d\omega \int_{-\infty }^{+\infty }\mathrm{dz}g_{\text{Qu}}^{*}\left(z\right)\frac{d}{\mathrm{dz}}g_{\text{Qu}}\left(z\right)\right\}$$

similar to the topological phases such as the Zak and Berry phases (*55*, *56*). Additional details on the derivation of χ including multiple electrons are detailed in text S3.

In the absence of phase matching, the terms $g_{\text{Qu}}$ and $g_{2}$ vanish. However, $g_{\phi }\neq 0$ because it is independent of the optical phase velocity. The phase χ is also nonzero. Therefore, the scattering operator in Eq. 3 reduces to

$$\hat{S}=e^{i\chi {\hat{N}}_{e}}e^{-ig_{\phi }{\hat{N}}_{e}\left(\hat{N}+\frac{1}{2}\right)}$$ (5)

where ${\hat{N}}_{e}=\sum_{k}{\hat{c}}_{k}^{†}{\hat{c}}_{k}$ is the electron number operator and $\hat{N}={\hat{a}}^{†}\hat{a}$ is the photon number operator. This photon-number–dependent scattering operator is our main result. In the rest of the article, the phase factors $e^{i\chi {\hat{N}}_{e}}$ and $e^{-\frac{1}{2}ig_{\phi }{\hat{N}}_{e}}$ are omitted because they are independent of any photonic excitation.

The scattering operator results in a dispersive interaction that counts electrons via the electron number operator ${\hat{N}}_{e}$. Its form as a dispersive Hamiltonian becomes more apparent using the undisturbed photonic Hamiltonian, ${\hat{H}}_{\text{ph}}$

$$\hat{S}=\text{exp}\left(-i\frac{g_{\phi }}{\text{ℏω}}{\hat{N}}_{e}{\hat{H}}_{\text{ph}}\right)$$

$\hat{S}$ commutes with each individual subsystem, $\left[\hat{S},{\hat{H}}_{e}\right]=\left[\hat{S},{\hat{H}}_{\text{ph}}\right]=0$. Thus, the interaction preserves the initial state up to a phase. Furthermore, the measured variable, ${\hat{N}}_{e}$, commutes with the undisturbed Hamiltonian ${\hat{H}}_{e}$, which satisfies the condition for QND (*50*–*52*, *57*–*59*) measurements. Thus, $\hat{S}$ constitutes a QND measurement of free-electron number. Next, we provide specific examples for the implication of Eq. 5 on states of a populated photonic mode.

### Electron-induced photonic phase shift

**Fig. 1.**
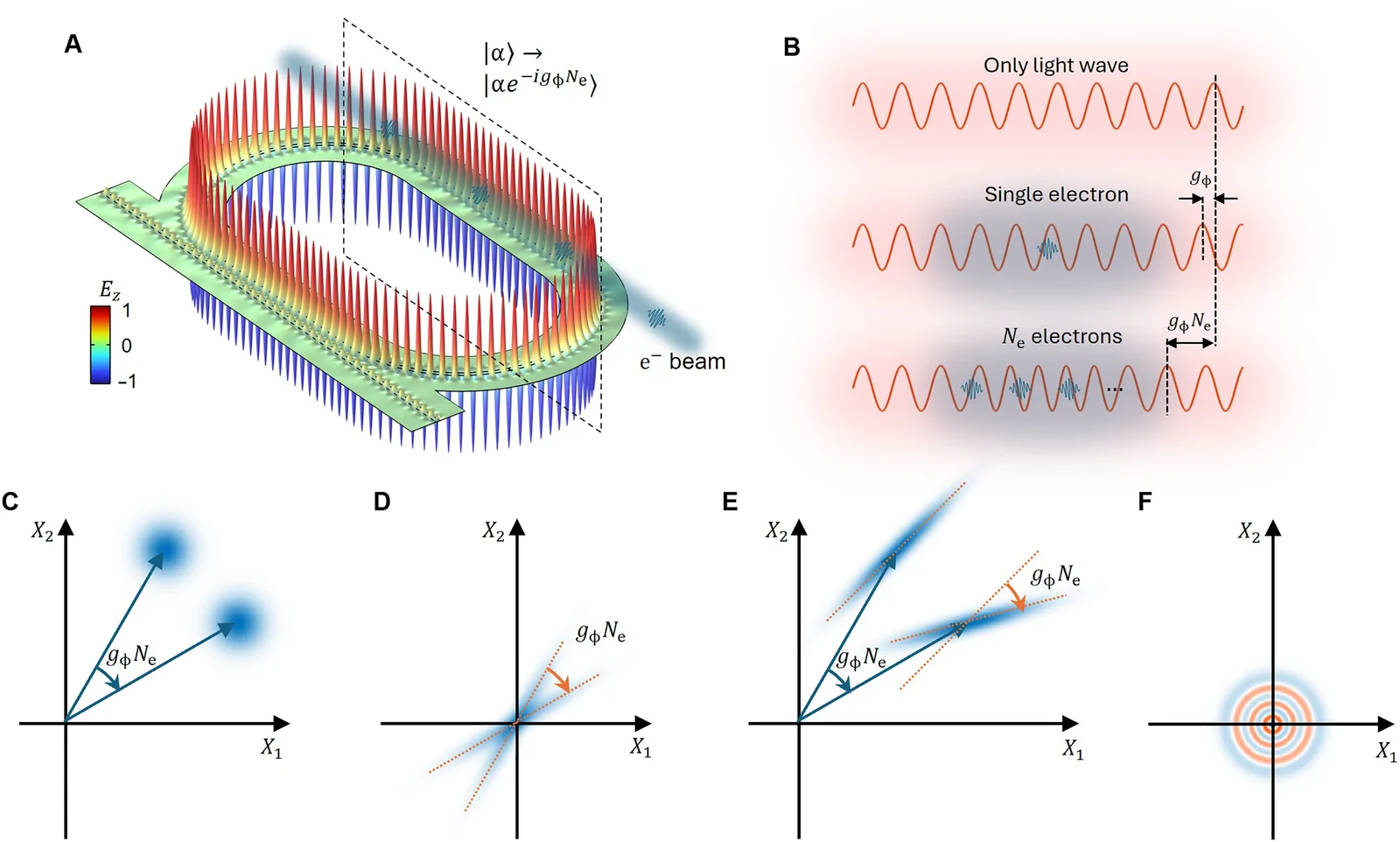
Optical phase shifts induced by elastically scattered free electrons. (**A**) A schematic shows the interaction of free electrons with an optical mode of a racetrack microresonator. The phase shift added to a coherent state ∣α⟩ is noted. (**B**) The elastic coupling constant, $g_{\phi }$, sets the proportion between the optical phase shift and the electron number, $N_{e}$. The red sinusoidal curves represent light waves, and the confined wave packet represent electrons. (**C** to **F**) Phase shifts of photonic states represented in phase space by their Wigner distribution: (C) coherent state, (D) squeezed vacuum state, (E) coherent squeezed state, and (F) non-Gaussian number state.

This section shows explicitly an electron-induced optical-phase shift for several important quantum-optical states. To have a concrete scenario in mind, consider the illustration in Fig. 1A, where electrons traverse the evanescent field of an optical mode of a microresonator. If the phase-matching terms are nullified by a mismatch between the velocities of the electrons and the optical phase, then the interaction is described by the QND scattering operator, $\hat{S}$.

Because the elastic scattering adds a pure phase for a system with precisely $N_{e}$ electrons, the resulting photonic state, $|\psi_{\text{ph},f}\rangle$, can be defined as the projection of the final state, $|\Psi_{f}\rangle =\hat{S}|\Psi_{i}\rangle$, on the initial electron state

$$|\psi_{\text{ph},f}\rangle =\left\langle \psi_{e}|\hat{S}|\Psi_{i}\right\rangle =e^{-i\frac{g_{\phi }}{\text{ℏω}}N_{e}{\hat{H}}_{\text{ph}}}|\psi_{\text{ph}}\rangle$$ (6)

See Materials and Methods for representation as a partial trace. By substituting the Schrödinger equation, ${\hat{H}}_{\text{ph}}=i\hbar \partial_{t}$, this photonic scattering operator can be represented as a time-shifting operator

$$\hat{S}=e^{\tau \partial_{t}}$$

This corresponds to propagating the photonic states by a time constant delay $\tau =\frac{1}{\omega }g_{\phi }N_{e}$. For modes with an angular frequency ω, it is a rotation $\text{ωτ}=g_{\phi }N_{e}$ in phase space. This optical phase is illustrated in Fig. 1B. If the initial photon state is a coherent state ∣α⟩, for $N_{e}$ electrons, $|\psi_{\text{ph},f}\rangle =|\alpha e^{-ig_{\phi }N_{e}}\rangle$, the optical state shifts

$$|\alpha \rangle \to |\alpha e^{-ig_{\phi }N_{e}}\rangle$$ (7)

The transformation adds a phase to the coherent-state parameter, which is a pure rotation in phase space, $g_{\phi }N_{e}$, as shown in Fig. 1C. In other words, a classical light field populating a single photonic mode will undergo a phase shift of $-g_{\phi }$ for every electron passage. For a beam with many electrons, the optical phase shift, δϕ, accumulates linearly, yielding a response proportional to the electron number (see details in Materials and Methods)

$$\text{δϕ}=-g_{\phi }N_{e}$$ (8)

In principle, this enables state-preserving electron counting by measuring laser phase shift, as schematically shown in Fig. 1B. In a later part, we provide for a specific proposal for a QND measurement of the number of electrons that pass within the mode’s coherence time.

One can apply the elastic scattering operator to additional photonic states. If the initial state is a squeezed vacuum ∣0,ζ⟩ (*60*), where ζ is the squeezing parameter, $\hat{S}$ applies

$$|0,\zeta \rangle \to |0,\zeta e^{-2ig_{\phi }N_{e}}\rangle$$ (9)

Detailed derivations are in Materials and Methods. The factor $e^{-2ig_{\phi }N_{e}}$ indicates that the free electrons can elastically rotate the squeezing direction clockwise with an angle $g_{\phi }N_{e}$, as shown in Fig. 1D. It implies that multiple electrons could rotate the squeezing direction by π/2.

For a coherent squeezed state ∣α,ζ⟩ (*49*, *61*), $\hat{S}$ induces the transformation

$$|\alpha ,\zeta \rangle \to |\alpha e^{-ig_{\phi }N_{e}},\zeta e^{-2ig_{\phi }N_{e}}\rangle$$

This is a combined result of Eqs. 7 and 9. Thus, the time shift induced by the elastic interaction allows an electron to impart a phase shift of the electric field of a coherent squeezed state and rotate the squeezing direction, as shown in Fig. 1E.

If the initial state is the joint state of two modes of coherent states, such as a cat state (*62*–*64*), assuming the elastic coupling strength of these two modes are equal

$$\left(|\alpha \rangle +e^{i\theta }|-\alpha \rangle \right)\to \left(|\alpha e^{-ig_{\phi }N_{e}}\rangle +e^{i\theta }|-\alpha e^{-ig_{\phi }N_{e}}\rangle \right)$$

which is the rotation in phase space around the origin for both modes.

If the initial photon state is a Fock state, ∣N⟩, the operator $\hat{S}$ just adds a phase, i.e.

$$|N\rangle \to e^{-ig_{\phi }N_{e}N}|N\rangle$$

equivalent to ωτ. In this trivial scenario, the interaction with the electron adds a pure global phase because the number state is a stationary state (Fig. 1F).

One can consider a N00N state, namely, a state with N photons in one or the other arm of an interferometer (*65*, *66*), where the free electron interacts in one of the arms only. The transformation is then

$$\frac{1}{\sqrt{2}}\left(|N\rangle |0\rangle +|0\rangle |N\rangle \right)\to \frac{1}{\sqrt{2}}\left(e^{-ig_{\phi }N_{e}N}|N\rangle |0\rangle +|0\rangle |N\rangle \right)$$

A subsequent interference between the two arms will oscillate proportionally to the multiplication $N_{e}N$.

### The electron-phase retarder as the classical limit

One expects that our quantum framework agrees with established theoretical and experimental results. Hence, we use it in the limit of a coherent optical state and reproduce the phase retardation that a laser induces with respect to noninteracting parts of the electron wave. Suppose the initial electron-photon state is $|\Psi_{i}\rangle =|\alpha \rangle ⊗2^{-1/2}\left(|\psi_{e}\rangle +|\psi_{r}\rangle \right)$. It includes an electron component that interacts with light, $|\psi_{e}\rangle$, a reference, $|\psi_{r}\rangle$, and a photonic coherent state, ∣α⟩, which overlaps one electron path. $|\Psi_{i}\rangle$ is subjected to a local elastic scattering operator in Eq. 5, yielding the final state, $|\Psi_{f}\rangle =\hat{S}|\Psi_{i}\rangle =2^{-1/2}\left(|\alpha e^{-ig_{\phi }{\hat{N}}_{e}}\rangle ⊗|\psi_{e}\rangle +|\alpha \rangle ⊗|\psi_{r}\rangle \right)$. Because the elastic coupling is typically weak, $g_{\phi }\ll 1$, the projection of the two photonic parts is a pure phase term, $\left\langle \alpha |\alpha e^{-ig_{\phi }{\hat{N}}_{e}}\right\rangle =\text{exp}\left({|\alpha |}^{2}\left(e^{-ig_{\phi }{\hat{N}}_{e}}-1\right)\right)\approx e^{-ig_{\phi }{|\alpha |}^{2}{\hat{N}}_{e}}$ (see Materials and Methods). Thus, in tracing out the photonic component, the final electron state is $2^{-1/2}\left(e^{-ig_{\phi }{|\alpha |}^{2}{\hat{N}}_{e}}|\psi_{e}\rangle +|\psi_{r}\rangle \right)$ (see Materials and Methods). The reference component was necessary to allow the partial trace to account for an otherwise immeasurable phase. From here on, we omit any referral to the reference for simplicity and mark the final electron state based only on the part that interacts with light, namely, $|\psi_{e,f}\rangle =e^{-ig_{\phi }{|\alpha |}^{2}{\hat{N}}_{e}}|\psi_{e}\rangle$. Substituting the mean photon number in the coherent state, ${|\alpha |}^{2}=\bar{N}$, we get the remaining electron state

$$|\psi_{e,f}\rangle =\prod_{k}e^{-ig_{\phi }\bar{N}{\hat{c}}_{k}^{†}{\hat{c}}_{k}}{\hat{c}}_{k}^{†}{|0\rangle }_{e}$$

That is, the phase of every traversing electron, ${\hat{c}}_{k}^{†}{|0\rangle }_{e}$, will be shifted by $-g_{\phi }\bar{N}$, thereby reproducing all the laser-induced electron-refraction phenomena: $\bar{N}$ accounts for the laser intensity and $g_{\phi }$ for the mode, interaction time, etc. For example, if the optical mode is a standing wave on the *x* axis, $g_{\phi }=g_{\phi }^{\text{max}}{\text{cos}}^{2}\left(2k_{\text{ph}}x+\theta \right)$, the light-induced phase is periodic. Here $g_{\phi }^{\text{max}}$ is the maximal elastic coupling, $k_{\text{ph}}$ is the photonic wave vector, and θ sets the peak position of the standing wave. A wide electron beam passing through would acquire this periodic spatial refraction, resulting in a Kapitza-Dirac diffraction. A narrow beam would acquire a phase between $e^{-ig_{\phi }^{\text{max}}\bar{N}}$ and zero, depending on its position (*40*). We note that the phase we omitted from Eq. 5, $e^{i\chi {\hat{N}}_{e}^{2}}$ and $e^{-\frac{1}{2}ig_{\phi }{\hat{N}}_{e}}$, is negligible in free space and could otherwise be made negligible for sufficiently strong optical pumping, $\bar{N}\gg 1$, because it does not scale with $\bar{N}$.

### The refractive index of free electrons

This section aims to assign a refractive index to a free electron, but in doing so, one needs to ask a fundamental question: What is the relevant scale on which to evaluate the electron? If it was a material, such as a nanoparticle positioned near the optical field, then it would induce refraction that depends on its radius, a property that the quantum electron lacks. Thus, we derive the refractive effect as a figure of merit and find its quantitative value. Furthermore, we express this electron refraction quantitatively using natural sizes of the system, namely, wavelength, volume, velocities, etc., and note the role played by the classical radius of a free electron.

To write the effective refractive index of the electron, we first rewrite the elastic coupling amplitude defined in Eq. 4 with $\Gamma =\text{diag}\left(1,1,\frac{1}{\gamma^{2}}\right)$. Assuming electrons propagate along *z* axis, and the transverse position, $r_{⊥}=\left(x,y\right)$, $g_{\phi }$ can be expressed as a function of the optical field integrated over the electron trajectory

$$g_{\phi }\left(r_{⊥}\right)=\frac{e^{2}}{\hbar p_{0}\omega^{2}}\int_{0}^{L}\mathrm{dz}\left({|E_{⊥}\left(r\right)|}^{2}+\frac{1}{\gamma^{2}}{|E_{z}\left(r\right)|}^{2}\right)$$ (10)

To estimate its figure of merit, we substitute the classical radius of the electron into the above equation, $r_{e}=\frac{e^{2}}{4\pi \epsilon_{0}m_{e}c^{2}}\approx 2.8\;\text{fm}$, such that its prefactor can be rewritten as $\frac{e^{2}}{p_{0}\hbar \omega^{2}}=\frac{2r_{e}}{\text{γβ}}\frac{\lambda_{0}\epsilon_{0}}{\hbar \omega }$. Here, $\epsilon_{0}$ is the vacuum permittivity, $\lambda_{0}=\frac{2\pi c}{\omega }$ is the optical wavelength in vacuum, and $E_{⊥},E_{z}$ are the electric field components perpendicular and parallel to the *z* axis. Assuming a mode volume V and, for now, a polarization perpendicular to the electron propagation direction, the electric field of one photon circulating in the cavity is $|E|=\sqrt{\frac{\hbar \omega }{2\epsilon_{0}V}}$. Thus, the elastic coupling strength reduces to a comparison between natural scales of the electron and optical quanta

$$g_{\phi }=\beta^{-1}\frac{r_{e}/\gamma }{V/\left(\lambda_{0}L\right)}=\frac{\text{electron radius}}{\text{photon}-\text{related width}}$$ (11)

**Fig. 2.**
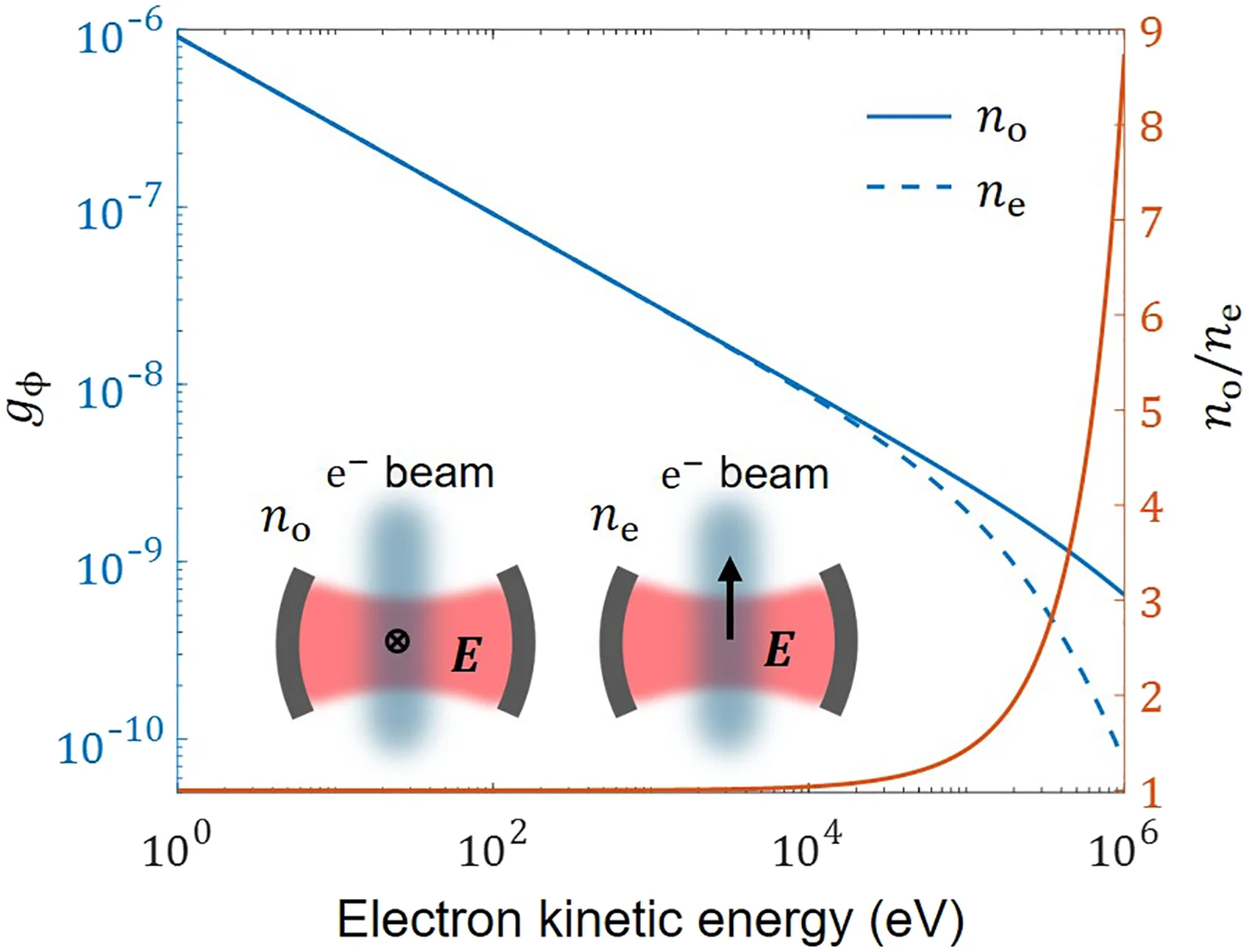
Energy scaling of the elastic electron-photon coupling. The refractive index contributed by free electrons becomes birefringent for kinetic energies above 10 keV. The sketches illustrate the ordinary and extraordinary polarization of the electron-induced birefringence. The numerical values here assume the light is confined in an optical cavity with a volume $V=\lambda_{0}^{3}$ and interaction length $L=\lambda_{0}=1550\;\text{nm}$.

$r_{e}/\gamma$ is the classical radius of the electron, subjected to a relativistic contraction, provides one length scale. The width related to the photon is $\frac{V}{\lambda_{0}L}$, which is the mode volume divided by the wavelength and interaction length. The inverse relativistic velocity parameter $\beta^{-1}=c/v_{0}$ scales the duration that a passing electron overlaps with the field. Because the classical radius of the electron is much smaller than the optical wavelength, this natural discrepancy of scales determines the weakness of the elastic interaction between an electron and a photon. For a photon scale of one wavelength, mode volume $V=\lambda_{0}^{3}$, interaction length $L=\lambda_{0}$, and $\lambda_{0}=1550\;\text{nm}$, $g_{\phi }$ is in the order of $10^{-6}$ to $10^{-10}$. Figure 2 shows the dependence of $g_{\phi }$ on kinetic energy of the electron.

In the description of classical electrodynamics, the mutual refraction of electrons and photons can be understood as particles passing through a medium that has an effective refractive index (*67*, *68*). Here, the change in refractive index, δn, can be inferred from electron-induced optical phase, by expressing the phase shift as the integral of the change of refractive index, $\text{δϕ}=\frac{2\pi }{\lambda_{0}}\int \mathrm{dz}\delta n$. Although arbitrary, one can use Eq. 8 to write $\delta n=-N_{e}\frac{\lambda_{0}}{2\pi }\frac{d}{\mathrm{dz}}g_{\phi }$, as derived in Materials and Methods. Furthermore, combining Eq. 10, we have

$$\delta n=-N_{e}\frac{e^{2}c}{p_{0}\hbar \omega^{3}}\left({|E_{⊥}|}^{2}+\frac{1}{\gamma^{2}}{|E_{z}|}^{2}\right)$$ (12)

Thus, the electron-induced δn is birefringent, governed by the birefringence matrix, Γ, and the overlap of the electron with the mode, $E_{⊥},E_{z}$. The optical axis is along the electron velocity. The ratio of ordinary and extraordinary refractive indices, $\gamma^{-2}$, as shown in Fig. 2, becomes substantial for kinetic energy scales of transmission EM, above 100 keV. The electron refractive index is proportional to $N_{e}$, the number of electrons that arrive within the lifetime of the optical mode, in agreement with our previous referral to the dispersive photonic Hamiltonian.

As a final note, in the simplified conceptual scenario where the interaction length $L=\lambda_{0}$, with uniformly distributed light fields, the change in refractive index can be rewritten as $\delta n=-N_{e}\frac{1}{2\pi }g_{\phi }$, with $g_{\phi }$ as in Eq. 11. In other words, the figure of merit for the electron’s refractive index is

$$\delta n=-\frac{N_{e}}{2\pi }\frac{\text{electron radius}}{\text{photon}-\text{related width}}$$

### Proposed experimental realization

This section addresses the challenge of free electron detection by measuring small phase shifts using interferometry with an optical cavity. We note that the use of optical resonators forces a trade-off among three timescales: the transition time, in which the electron modifies the optical refractive index; the cavity lifetime, which sets the rate of information leakage out to a detector; and the detector response time, which is implicitly integrated over. The motivation for using microresonators is the prospect of superb spectral precision that may enable the sensitivity necessary for a QND detection down to a single electron, i.e., exact counting. Furthermore, high-quality optical resonators can be fabricated with various dielectric materials and geometrical shapes (*69*–*71*).

**Fig. 3.**
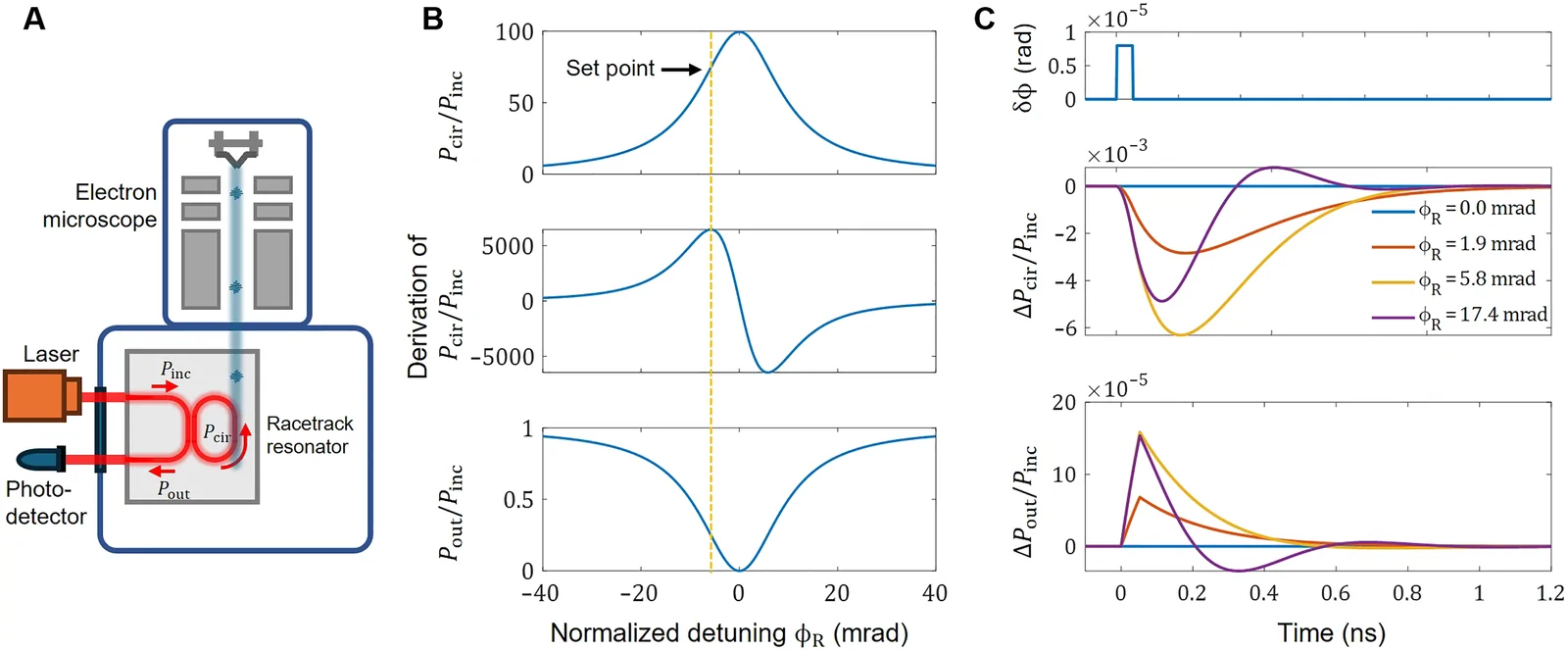
A proposal for measuring electron-induced phase shifts in an optical microresonator. (**A**) Schematic outline of a setup for counting free electrons by measuring the energy output of a resonator. The optical phase shift of light circulating in the resonator due to electron passages perturbs power measured in an ideal photodetector. (**B**) Side-of-fringe locking approach for a resonator with parameters r=a=0.995. The detuning is represented by the round-trip phase, $\phi_{R}$. The circulating power, its derivative with respect to the detuning and the output power are presented for a high-finesse cavity on the top, middle, and bottom, respectively. The dashed line marks the setting point, where the output power depends maximally on small (electron-induced) retardation. (**C**) Dynamics of the circulating and output power following a simplistic binary (on-off) dispersion induced by one electron, assuming an interaction time of 53 ps with $g_{\phi }=8\times 10^{-6}$ radians (top). The cavity has a round-trip duration of $T_{R}=$2 ps and a lifetime of 200 ps, which for a wavelength of 1550 nm corresponds to a quality factor of $∼2.4\times 10^{5}$. The resulting transient decrease of the circulating power and increase of the output power are plotted on the middle and bottom, respectively, for various initial detuning set points, $\phi_{R}$.

Figure 3A outlines the schematics of QND measurement in an EM. A racetrack resonator, also shown in fig. S1, is placed in the vacuum chamber and connected with input/output fibers. If a circulating optical mode is strongly confined, then an electron-induced intraresonator phase shift changes the output power. For a high finesse (F) racetrack resonator, which is available in modern micro/nanofabrication technologies (*72*, *73*), the power in the racetrack ($P_{\text{cir}}$) can be enhanced by several orders of magnitude compared with the input power ($P_{\text{inc}}$). The enhancement factor (see Fig. 3B) depends on phase accumulated in one round trip (*74*), $\phi_{R}=T_{R}\omega$, where $T_{R}$ is the duration of one round trip. An intracavity phase added dynamically by a traversing electron is equivalent to a time-varying detuning. The outcoupled power is sensitive to $\phi_{R}$ when the cavity is pumped on the side of the resonance peak. At the half maximum of the resonance peak, the enhancement factor is approximately $F^{2}/\pi^{2}$ (see text S4).

Because the effect induced by the electron is transient, we turn to a dynamic analysis of the optical effects induced in the cavity. The energy variation in the cavity depends on two of its timescales, the round-trip time ($T_{R}$) and the ringdown time (or lifetime). For simplicity, the light is assumed as a single mode, and the electron is assumed to be slow, such that its transition is longer than $T_{R}$ and its refractive effect is enhanced. We refer to the electron’s passage as time zero and analyze the light in the cavity in subsequent round trips. The full expressions of the circulating and output fields with respect to their steady state are derived in text S5 and only outlined here. Consider the optical amplitude and phase accumulated in a round trip, expressed as $x=\mathrm{ra}e^{i\phi_{R}}$, r is the coefficient of light to remain in resonator, and a is the coupling to the output port, such that $\left(1-a^{2}\right)$ is the absorption in one round trip (*74*). An abrupt phase shift in the resonator generates a circulating phase-shifted pulse, ringing with intervals $T_{R}$. The *m*th ringing event has a complex amplitude $x^{m}$, which is decaying per round trip. After time t, the effects of the phase shift accumulate to $\Sigma_{m=0}^{+\infty }x^{m+1}\left(e^{i\text{δϕ}\left(t-mT_{R}\right)}-1\right)$. In typical resonators, the light rings multiple times before it radiates, whereas the cavity is also repumped. The dynamics of several parameters is shown in Fig. 3C. All the parameters stabilize after few cavity lifetimes. The circulating power decreases at first and then recovers to a steady state due to refilling by the pump. The output power initially increases before stabilization, and it is this variation that one should detect to count the electrons. In other words, an ideal detector should have sufficient bandwidth to discern this deviation from a steady state. We note that the optical dynamics following the case of an abrupt electron transition is more involved. Briefly, when the interaction is shorter than $T_{R}$, the induced refraction does not affect the entire circulating wave, so the output includes many weak discrete echoes of the pulse. Figure S2 illustrates such a case, assuming a simplistic rectangular phase kick and ignoring dispersion effects, for analytic clarity.

For an analysis of the circulating and output power, we consider a pump detuned to the steepest point of the resonance peak. The net energy output due to the phase shift is proportional to the first-order derivative of the circulating power and the energy absorption in the resonator, $E_{\text{net}}\propto \left(1-a^{2}\right)\frac{d}{d\omega }P_{\text{cir}}\left(\omega \right)$. In a lossless cavity (a=1), the effect nullifies. To calculate a concrete example and evaluate the electron-counting sensitivity, we assume that the electron beam induces a constant phase shift, δϕ, for a duration of the integration time, $T_{\text{int}}$. The energy output in a high-finesse microresonator is (the detailed derivation is in text S6)

$$E_{\text{net}}\approx -\frac{1}{2}P_{\text{cir}}\cdot T_{\text{int}}\cdot \text{δϕ}$$ (13)

Therefore, the net energy output for a single electron passage is proportional to the circulating power. If we consider a racetrack microresonator with a 100-μm-long straight segment and a cross-sectional area only 0.037$\lambda_{0}^{2}$ (such as silicon waveguide with typical cross section of 220 nm by 400 nm and a wavelength of 1550 nm), a circulating power of 300 mW, and an electron with a kinetic energy of 10 eV, then interaction time is $T_{\text{int}}=53\;\text{ps}$ and $g_{\phi }\approx 8\times 10^{-6}$. In such an example, the change of refractive index would be $\delta n\approx -2\times 10^{-8}$. The net energy output from the resonator according to Eq. 13 is ∼$6.3\times 10^{-17}$ joules. This value is approximately the energy of 500 photons per passing electron, which is similar to the shot noise fluctuations of the output coherent state. One can ask how to optimize the sensitivity of the nondestructive counting, which is an important outcome of this work. Hence, we expand the analysis of the signal-to-noise ratio of the photonic measurement versus the normalized detuning ($\phi_{R}$) in text S7 and fig. S3. We evaluate the reduction of the electron number variance in text S8 and fig. S4. On the basis of that analysis, the specific parameters given in the example should suffice for the detection of a few electrons. One can deduce that single-electron sensitivity requires a high circulating power, long interaction duration, and probably, an optimized optical resonator. Furthermore, the choice of the optical cavity will have to address additional technical issues, such as transverse deflections of the electron induced by the optical field (see details in text S9).

## DISCUSSION

This work establishes a comprehensive quantum framework for the elastic interaction of free electrons and photons, which puts both particles on an equal footing. Whereas intense laser fields are known to induce electron-phase retardation, we find the fundamental back action that is hidden when light is addressed classically, namely, we quantify photonic phase retardation induced by a traversing electron. A free electron adds a refractive index onto the light field, with nontrivial properties such as birefringent optical axis aligned with the electron velocity. We addressed several properties of this back action, showing that it scales linearly with the number of electrons. We detail its operation on various optical fields, such as coherent states, squeezed states, Fock states, and N00N states.

Because the electron-induced refraction constitutes a QND estimator for the electron number, we propose it as a state-preserving approach for counting electrons, which could enable sub-Poissonian electron distribution. We provide a preliminary example for such a counting experiment to emphasize parameters one should consider when aiming at a single-electron sensitivity. Thus, in addition to the fundamental properties of dispersive electron-photon coupling presented here, the quantum electron states that elastic coupling would enable may bring signal-to-noise ratio in electron microscopy to surpass the standard quantum limit (*65*, *75*, *76*) and, generally, empower quantum sensing with electrons, with techniques reserved so far for quantum optics.

## MATERIALS AND MATERIALS AND METHODS

### Final photonic state

Assuming an arbitrary photonic state, $|\psi_{\text{ph}}\rangle$, the initial state is $|\Psi_{i}\rangle =|\psi_{\text{ph}}\rangle ⊗|\psi_{e}\rangle$, and the final is $|\Psi_{f}\rangle =\hat{S}\left(|\psi_{\text{ph}}\rangle ⊗|\psi_{e}\rangle \right)$. Thus

$${\hat{\rho }}_{f}=|\Psi_{f}\rangle \langle \Psi_{f}|=\left(\hat{S}|\psi_{e}\rangle |\psi_{\text{ph}}\rangle \langle \psi_{\text{ph}}|\langle \psi_{e}|{\hat{S}}^{†}\right)$$

For a state with $N_{e}$ electrons, $|\Psi_{f}\rangle =\hat{S}|\psi_{e}\rangle |\psi_{\text{ph}}\rangle =|\psi_{e}\rangle e^{-i\frac{g_{\phi }}{\hbar \omega }N_{e}{\hat{H}}_{\text{ph}}}|\psi_{\text{ph}}\rangle$

$${\hat{\rho }}_{f}=|\Psi_{f}\rangle \langle \Psi_{f}|=\left(|\psi_{e}\rangle e^{-i\frac{g_{\phi }}{\text{ℏω}}N_{e}{\hat{H}}_{\text{ph}}}|\psi_{\text{ph}}\rangle \langle \psi_{\text{ph}}|e^{i\frac{g_{\phi }}{\text{ℏω}}N_{e}{\hat{H}}_{\text{ph}}}\langle \psi_{e}|\right)$$

A partial trace on the electronic component

$${\text{Tr}}_{e}\left({\hat{\rho }}_{f}\right)=\left(e^{-i\frac{g_{\phi }}{\text{ℏω}}N_{e}{\hat{H}}_{\text{ph}}}|\psi_{\text{ph}}\rangle \langle \psi_{\text{ph}}|e^{-i\frac{g_{\phi }}{\text{ℏω}}N_{e}{\hat{H}}_{\text{ph}}}\langle \psi_{e}||\psi_{e}\rangle \right)=|\psi_{\text{ph},f}\rangle \langle \psi_{\text{ph},f}|$$

Hence, the final photonic state is $|\psi_{\text{ph},f}\rangle =e^{-i\frac{g_{\phi }}{\hbar \omega }N_{e}{\hat{H}}_{\text{ph}}}|\psi_{\text{ph}}\rangle =\left\langle \psi_{e}|\hat{S}|\Psi_{i}\right\rangle$, as in the Eq. (6).

### The phase shift of a coherent state

If the initial photonic state is a coherent state, using $e^{-ig_{\phi }\hat{N}}|\alpha \rangle =|\alpha e^{-ig_{\phi }}\rangle$, then we have the final state $|\Psi_{f}\rangle =\hat{S}|\alpha \rangle ⊗|\psi_{e}\rangle =e^{-\frac{1}{2}ig_{\phi }{\hat{N}}_{e}}|\alpha e^{-ig_{\phi }{\hat{N}}_{e}}\rangle ⊗|\psi_{e}\rangle$. Then, the final photonic state is $\left\langle \psi_{e}|\Psi_{f}\right\rangle =e^{-\frac{1}{2}ig_{\phi }N_{e}}|\alpha e^{-ig_{\phi }N_{e}}\rangle$.

To confirm that the phase shift of the coherent field is $\text{δϕ}=-g_{\phi }N_{e}$, we evaluate the expectation value of the electric field operator as follows. Taking the usual single-mode electric-field operator, $\hat{E}\left(t\right)=E^{*}\left(r\right){\hat{a}}^{†}e^{i\omega t}+E\left(r\right)\hat{a}e^{-i\omega t}$ into the calculation, the expectation value of the electrical field in this phase-shift coherent state is $\left\langle \hat{E}\left(t\right)\right\rangle =\left\langle \alpha e^{-ig_{\phi }N_{e}}|\hat{E}\left(t\right)|\alpha e^{-ig_{\phi }N_{e}}\right\rangle$. Here, the factor $e^{-\frac{1}{2}ig_{\phi }N_{e}}$ drops out of expectation values. For the expectation value of annihilation and creation operators, we have $\left\langle \alpha e^{-ig_{\phi }N_{e}}|\hat{a}|\alpha e^{-ig_{\phi }N_{e}}\right\rangle =\alpha e^{-i\omega t-ig_{\phi }N_{e}}$, and the electric-field expectation-value becomes

$$\left\langle \hat{E}\left(t\right)\right\rangle =2\text{Re}\left(E\left(r\right)\alpha e^{-i\omega t-ig_{\phi }N_{e}}\right)$$

Thus, it can be confirmed that the additional phase in the electric field is $\text{δϕ}=-g_{\phi }N_{e}$.

### The phase shift of a squeezed state

For a coherent squeezed state, to prove $e^{-ig_{\phi }\hat{N}}|\alpha ,\zeta \rangle =|\alpha e^{-ig_{\phi }},\zeta e^{-2ig_{\phi }}\rangle$, recall that a coherent squeezed state can be written as $|\alpha ,\zeta \rangle =\hat{D}\left(\alpha \right)\hat{𝒮}\left(\zeta \right)|0\rangle$, where $\hat{D}\left(\alpha \right)=e^{\alpha {\hat{a}}^{†}-\alpha^{*}\hat{a}}$ is the displacement operator and $\hat{𝒮}\left(\zeta \right)=e^{\frac{1}{2}\left(\zeta^{*}{\hat{a}}^{2}-\zeta {\hat{a}}^{†2}\right)}$ is the squeezing operator.

Let us first analyze the transformation of the annihilation operator $\hat{a}$ under conjugation by the phase shift operator $e^{-ig_{\phi }\hat{N}}$. Using the Baker-Campbell-Hausdorff formula, we have $e^{-ig_{\phi }\hat{N}}\hat{a}e^{ig_{\phi }\hat{N}}=\hat{a}+ig_{\phi }\hat{a}+\frac{1}{2!}{\left(ig_{\phi }\right)}^{2}\hat{a}+\ldots =e^{ig_{\phi }}\hat{a}$.

Similarly, we obtain $e^{-ig_{\phi }\hat{N}}{\hat{a}}^{2}e^{ig_{\phi }\hat{N}}=e^{-ig_{\phi }\hat{N}}\hat{a}e^{ig_{\phi }\hat{N}}e^{-ig_{\phi }\hat{N}}\hat{a}e^{ig_{\phi }\hat{N}}=e^{2ig_{\phi }}{\hat{a}}^{2}$. The transformation of creation operator: $e^{-ig_{\phi }\hat{N}}{\hat{a}}^{†}e^{ig_{\phi }\hat{N}}=e^{-ig_{\phi }}{\hat{a}}^{†}$, $e^{-ig_{\phi }\hat{N}}{\hat{a}}^{†2}e^{ig_{\phi }\hat{N}}=e^{-2ig_{\phi }}{\hat{a}}^{†2}$.

Now, consider the transformation of the displacement operator: $e^{-ig_{\phi }\hat{N}}\hat{D}\left(\alpha \right)e^{ig_{\phi }\hat{N}}=\text{exp}\left(\alpha e^{-ig_{\phi }}{\hat{a}}^{†}-\alpha^{*}e^{ig_{\phi }}\hat{a}\right)=\hat{D}\left(\alpha e^{-ig_{\phi }}\right)$. The transformation of the squeezing operator: $e^{-ig_{\phi }\hat{N}}\hat{𝒮}\left(\zeta \right)e^{ig_{\phi }\hat{N}}=\text{exp}\frac{1}{2}\left(\zeta^{*}e^{2ig_{\phi }}{\hat{a}}^{2}-\zeta e^{-2ig_{\phi }}{\hat{a}}^{†2}\right)=\hat{𝒮}\left(\zeta e^{-2ig_{\phi }}\right)$.

Last, applying the transformed operators to the coherent squeezed state: $e^{-ig_{\phi }\hat{N}}|\alpha ,\zeta \rangle =e^{-ig_{\phi }\hat{N}}\hat{D}\left(\alpha \right)\hat{𝒮}\left(\zeta \right)|0\rangle =e^{-ig_{\phi }\hat{N}}\hat{D}\left(\alpha \right)e^{ig_{\phi }\hat{N}}e^{-ig_{\phi }\hat{N}}\hat{𝒮}\left(\zeta \right)e^{ig_{\phi }\hat{N}}e^{-ig_{\phi }\hat{N}}|0\rangle =\hat{D}\left(\alpha e^{-ig_{\phi }}\right)\hat{𝒮}\left(\zeta e^{-2ig_{\phi }}\right)|0\rangle =|\alpha e^{-ig_{\phi }},\zeta e^{-2ig_{\phi }}\rangle$. Thus, we have the transformation of coherent squeezed state. Let α=0, then we have the transformation of the squeezed vacuum state.

### The phase shift of the electron wave function

To prove $\left\langle \alpha |\alpha e^{-ig_{\phi }{\hat{N}}_{e}}\right\rangle \approx e^{-ig_{\phi }{|\alpha |}^{2}{\hat{N}}_{e}}$, one can expand the coherent state in the basis of number states, that is, $|\alpha e^{-ig_{\phi }{\hat{N}}_{e}}\rangle =e^{-\frac{{|\alpha |}^{2}}{2}}\sum_{n=0}^{\infty }\frac{1}{\sqrt{n!}}$. Substituting ⟨α∣ and $|\alpha e^{-ig_{\phi }{\hat{N}}_{e}}\rangle$ with the expressions in number state basis gives

$$\begin{array}{c} \left\langle \alpha |\alpha e^{-ig_{\phi }{\hat{N}}_{e}}\right\rangle =\left(e^{-\frac{{|\alpha |}^{2}}{2}}\sum_{m=0}^{\infty }\frac{1}{\sqrt{m!}}{\left(\alpha^{*}\right)}^{m}\langle m|\right)\\ \left(e^{-\frac{{|\alpha |}^{2}}{2}}\sum_{n=0}^{\infty }\frac{1}{\sqrt{n!}}{\left(\alpha e^{-ig_{\phi }{\hat{N}}_{e}}\right)}^{n}|n\rangle \right)=e^{-{|\alpha |}^{2}}\sum_{n=0}^{\infty }\frac{1}{n!}{|\alpha |}^{2n}{\left(e^{-ig_{\phi }{\hat{N}}_{e}}\right)}^{n}\\ =e^{-{|\alpha |}^{2}}e^{{|\alpha |}^{2}e^{-ig_{\phi }{\hat{N}}_{e}}}=\text{exp}\left({|\alpha |}^{2}\left(e^{-ig_{\phi }{\hat{N}}_{e}}-1\right)\right) \end{array}$$

Because $g_{\phi }\ll 1$, we have $e^{-ig_{\phi }{\hat{N}}_{e}}\approx 1-ig_{\phi }{\hat{N}}_{e}$. Thus $\left\langle \alpha |\alpha e^{-ig_{\phi }{\hat{N}}_{e}}\right\rangle \approx e^{-ig_{\phi }{|\alpha |}^{2}{\hat{N}}_{e}}$.

For the final system state $|\Psi_{f}\rangle =2^{-1/2}\left(|\alpha e^{-ig_{\phi }{\hat{N}}_{e}}\rangle ⊗|\psi_{e}\rangle +|\alpha \rangle ⊗|\psi_{r}\rangle \right)$, the density matrix is ${\hat{\rho }}_{f}=|\Psi_{f}\rangle \langle \Psi_{f}|=\frac{1}{2}\left(|\alpha e^{-ig_{\phi }{\hat{N}}_{e}}\rangle \right.\langle \alpha e^{-ig_{\phi }{\hat{N}}_{e}}||\psi_{e}\rangle$, and the partial density matrix is

$Tr_{\text{ph}}\left({\hat{\rho }}_{f}\right)=\frac{1}{2}(|\psi_{e}\rangle \langle \psi_{e}|+\left\langle \alpha |\alpha e^{-ig_{\phi }{\hat{N}}_{e}}\right\rangle |\psi_{e}\rangle \langle \psi_{r}|+\left\langle \alpha e^{-ig_{\phi }{\hat{N}}_{e}}|\alpha \right\rangle |\psi_{r}\rangle \langle \psi_{e}|+|\psi_{r}\rangle \langle \psi_{r}|)=\frac{1}{2}\left(e^{-ig_{\phi }{|\alpha |}^{2}{\hat{N}}_{e}}|\psi_{e}\rangle +|\psi_{r}\rangle \right)\left(e^{ig_{\phi }{|\alpha |}^{2}{\hat{N}}_{e}}\langle \psi_{e}|+\langle \psi_{r}|\right)$. Thus, the final electron state is $2^{-1/2}\left(e^{-ig_{\phi }{|\alpha |}^{2}{\hat{N}}_{e}}|\psi_{e}\rangle +|\psi_{r}\rangle \right)$.

### The change of refractive index

If we consider l to be the total light-propagation length and L to be the electron-photon interaction length, then the total light phase can be expressed as the path integral of refractive index, $\phi +\text{δϕ}=\frac{2\pi }{\lambda_{0}}\left(\mathrm{nl}+\int_{0}^{L}\mathrm{dz}\delta n\right)$. Thus, the additional phase, δϕ, induced by a free electron, is the path integral of the change of the refractive index, $\text{δϕ}=\frac{2\pi }{\lambda_{0}}\int_{0}^{L}\mathrm{dz}\delta n$. In differential form, $\delta n=\frac{\lambda_{0}}{2\pi }\frac{d}{\mathrm{dz}}\text{δϕ}$. Combining with Eq. 8, that is, $\text{δϕ}=-g_{\phi }N_{e}$, then we have $\delta n=-N_{e}\frac{\lambda_{0}}{2\pi }\frac{d}{\mathrm{dz}}g_{\phi }$.

## Supplementary Materials

### This PDF file includes:

Supplementary Text S1 to S9 Figs. S1 to S4 References
