# Supplementary material for: Elastic quantum coupling between free electrons and photons
Source: Sci Adv. 2026 Sep 4;12(36):eaed3278. doi: 10.1126/sciadv.aed3278 (PMC13544211; doi:10.1126/sciadv.aed3278)
Supplement: Supplementary file 1 [file sciadv.aed3278_sm.pdf]

Science Advances
AAAS

Supplementary Materials for

Elastic quantum coupling between free electrons and photons

Dingguo Zheng and Ofer Kfir

Corresponding author: Ofer Kfir, kfir@tauex.tau.ac.il

Sci. Adv. **12**, eaed3278 (2026)
DOI: 10.1126/sciadv.aed3278

This PDF file includes:

Supplementary Text S1 to S9
Figs. S1 to S4
References

S1. The Hamiltonian of free-electron-photon system

The free-electron-photon interaction Schrödinger equation has been treated in several forms in the literature. For first-order coupling (interaction Hamiltonian proportional to vector potential $\boldsymbol{A}$), solutions have been demonstrated in the many works on quantum photon-induced near-field electron microscopy (*4*, *23*, *28*, *32*). For second-order coupling (proportional to $\boldsymbol{A}^2$), related treatments appear in studies of laser manipulation of electron beams (*44*, *53*, *45*, *77*). However, a fully quantum theory that treats both the free electron and photon subsystems and includes second-order interactions remain incomplete.

To illustrate the effect of free-electron-photon interaction on the photon subsystem, we begin with a free electron of momentum $\boldsymbol{p}_0$ and rest energy $m_\mathrm{e}c^2$ propagating in free space. A concrete example is the flying free electrons as probes in electron microscopes. The relativistic energy of electron is

$$\varepsilon = \sqrt{m_\mathrm{e}^2 c^4 + p^2 c^2}. \tag{S1}$$

To expand the electron energy about the initial energy $\varepsilon_0 = \sqrt{m_\mathrm{e}^2 c^4 + {p_0}^2 c^2}$, define the momentum change $\Delta\boldsymbol{p} = \boldsymbol{p} - \boldsymbol{p}_0$. Substituting $\boldsymbol{p}_0 + \Delta\boldsymbol{p}$ into Eq. (S1) yields

$$\varepsilon = \varepsilon_0 \sqrt{1 + \frac{2}{\varepsilon_0}\boldsymbol{v}_0 \cdot \Delta\boldsymbol{p} + \frac{c^2}{\varepsilon_0^2}(\Delta\boldsymbol{p}) \cdot (\Delta\boldsymbol{p})}.$$

where $\boldsymbol{v}_0 = \boldsymbol{p}_0 c^2/\varepsilon_0$ is electron initial group velocity. Using formula $\sqrt{1+x} = 1 + \frac{x}{2} - \frac{x^2}{8} + \cdots$, we expand $\varepsilon = (\Delta\boldsymbol{p})$ to second-order in $\Delta\boldsymbol{p}$ and obtain

$$\varepsilon(\Delta\boldsymbol{p}) = \varepsilon_0 + \boldsymbol{v}_0 \cdot \Delta\boldsymbol{p} + \frac{1}{2\gamma m_\mathrm{e}}(\Delta\boldsymbol{p})^\mathrm{T}\boldsymbol{\Gamma}(\Delta\boldsymbol{p}) + \cdots \tag{S2}$$

where $\boldsymbol{\Gamma}$ is a 3×3 matrix defined as

$$\boldsymbol{\Gamma} = I_3 - \frac{\boldsymbol{v_0}}{c}\frac{\boldsymbol{v_0}^\mathrm{T}}{c},$$

I_3 is a 3×3 identity matrix. If electrons propagate along z-direction, then $\boldsymbol{v_0} = (0,0,v_0)^\mathrm{T}$, $\boldsymbol{\Gamma}$ reduce to a diagonal matrix and $\boldsymbol{\Gamma} = \mathrm{diag}(1,1,\frac{1}{\gamma^2})$.

On the other hand, consider a monochromatic light propagating in vacuum or an optical microcavity. According to the theory of quantum optics, the vector potential of light can be written as

$$\widehat{\boldsymbol{A}} = \boldsymbol{A}\hat{a}\mathrm{e}^{-\mathrm{i}\omega t} + \boldsymbol{A}^*\hat{a}^\dagger\mathrm{e}^{\mathrm{i}\omega t}, \tag{S3}$$

where $\boldsymbol{A} = -\mathrm{i}\frac{1}{\omega}\boldsymbol{E}(\boldsymbol{r})$, $\boldsymbol{A}^* = \mathrm{i}\frac{1}{\omega}\boldsymbol{E}^*(\boldsymbol{r})$, ω is light frequency, $\hat{a}$ ($\hat{a}^\dagger$) is photon annihilation (creation) operator, $\boldsymbol{E}(\boldsymbol{r})$ is the electric field of single photon. The Hamiltonian of light is

$$\widehat{H}_\mathrm{ph} = \hbar\omega\left(\hat{a}^\dagger\hat{a} + \frac{1}{2}\right) \tag{S4}$$

In the free-electron-photon interaction, consider electrons and photons as a combined system that the joint state can be written as $|\psi_\mathrm{ph}\rangle \otimes |\psi_\mathrm{e}\rangle$. Working at minimal coupling scheme, the momentum $\boldsymbol{p}$ can be replaced by mechanical momentum operator $\hat{p} + e\widehat{\boldsymbol{A}}$, where $\hat{p} = -\mathrm{i}\hbar\nabla$ is the momentum operator, e is elementary charge. Since there are no charges or currents in the path of the electron, the scalar potential of electromagnetic wave is zero. Ignoring the spin of electron, taking the expression of $\Delta\boldsymbol{p} = \hat{p} - \boldsymbol{p}_0 + e\widehat{\boldsymbol{A}}$ into Eq. (S2), we have the Hamiltonian of electron-photon combined system,

$$\hat{H} \cong \hat{H}_{\mathrm{ph}} + \varepsilon_0 + \boldsymbol{v}_0 \cdot \left(\hat{p} - \boldsymbol{p}_0 + e\hat{\boldsymbol{A}}\right) + \frac{1}{2\gamma m_{\mathrm{e}}}\left(\hat{p} - \boldsymbol{p}_0 + e\hat{\boldsymbol{A}}\right)^{\mathrm{T}} \boldsymbol{\Gamma} \left(\hat{p} - \boldsymbol{p}_0 + e\hat{\boldsymbol{A}}\right). \tag{S5}$$

Eq. (S5) describes the Hamiltonian for a single electron interacting with the light field. To describe multiple electrons, we quantize the electron field using the fermion annihilation (creation) operator $\hat{c}_k$ ($\hat{c}_k^\dagger$), which satisfy the anticommutation relation

$$\{\hat{c}_{k'}, \hat{c}_k^\dagger\} = \delta_{k',k}, \{\hat{c}_{k'}, \hat{c}_k\} = \{\hat{c}_{k'}^\dagger, \hat{c}_k^\dagger\} = 0.$$

The Hamiltonian can then be written as the sum of a non-interaction part and an interaction part,

$$\hat{H} = \hat{H}_0 + \hat{H}_{\mathrm{int}}, \tag{S6}$$

where

$$\hat{H}_0 = \hat{H}_{\mathrm{ph}} + \hat{H}_{\mathrm{e}}. \tag{S7}$$

$\hat{H}_{\mathrm{ph}}$ is defined in Eq. (S4) and the non-interaction Hamiltonian of electrons

$$\hat{H}_{\mathrm{e}} = \sum_{k=k_1}^{k_{N_{\mathrm{e}}}} \hat{\varepsilon}_k \hat{c}_k^\dagger \hat{c}_k \tag{S8}$$

where ε_k is single electron Hamiltonian. According to Eq. (S5),

$$\hat{\varepsilon}_k = \varepsilon_{0,k} + \boldsymbol{v}_{0,k} \cdot \left(\hat{p} - \boldsymbol{p}_{0,k}\right) + \frac{1}{2\gamma_k m_{\mathrm{e}}}\left(\hat{p} - \boldsymbol{p}_{0,k}\right)^{\mathrm{T}} \boldsymbol{\Gamma} \left(\hat{p} - \boldsymbol{p}_{0,k}\right).$$

Since we focus on the phase-mismatched interaction, the electron-energy variations during the interaction is small. Therefore, the second-order term in $\hat{\varepsilon}_k$ presenting the square of momentum changes can be neglected. Furthermore, if we consider electrons have the same initial momentum, then $\varepsilon_{0,k} = \varepsilon_0$, $\boldsymbol{v}_{0,k} = \boldsymbol{v}_0$, $\boldsymbol{p}_{0,k} = \boldsymbol{p}_0$, $\hat{\varepsilon}_k$ reduce to

$$\hat{\varepsilon}_k = \varepsilon_0 + \boldsymbol{v}_0 \cdot (\hat{p} - \boldsymbol{p}_0). \tag{S9}$$

The interaction Hamiltonian

$$\hat{H}_{\mathrm{int}} = \sum_{k=k_1}^{k_{N_e}} \left(e\boldsymbol{v}_0 \cdot \hat{\boldsymbol{A}} + \frac{e^2}{2\gamma m_{\mathrm{e}}} \hat{\boldsymbol{A}}^{\mathrm{T}} \boldsymbol{\Gamma} \hat{\boldsymbol{A}} + \hat{H}_{pA}\right) \hat{c}_k^\dagger \hat{c}_k \tag{S10}$$

where coupling term $\hat{H}_{pA}$ is linear with vector potential and dependent on the changes of momentum,

$$\hat{H}_{pA} = \frac{e}{2\gamma m_{\mathrm{e}}}\left((\hat{p} - \boldsymbol{p}_0)^{\mathrm{T}} \boldsymbol{\Gamma} \hat{\boldsymbol{A}} + \hat{\boldsymbol{A}}^{\mathrm{T}} \boldsymbol{\Gamma} (\hat{p} - \boldsymbol{p}_0)\right).$$

Choose Coulomb gauge ($\nabla \cdot \boldsymbol{A} = 0$), $\hat{H}_{pA}$ can be reduced to $\frac{e}{\gamma m_{\mathrm{e}}} \hat{\boldsymbol{A}}^{\mathrm{T}} \boldsymbol{\Gamma} (\hat{p} - \boldsymbol{p}_0)$. Since the changes of momentum are small during phase-mismatching interaction, we neglect $\hat{H}_{pA}$ in the following calculation. Inserting Eq. (S3) into Eq. (S10) and rearrange, we have the interaction Hamiltonian

$$\hat{H}_{\mathrm{int}} = \sum_{k=k_1}^{k_{N_{\mathrm{e}}}} \left(\hat{H}_1 + \hat{H}_2 + \hat{H}_\phi\right) \hat{c}_k^\dagger \hat{c}_k\,, \tag{S11}$$

where the expressions of $\hat{H}_1$, $\hat{H}_2$ and $\hat{H}_\phi$ are

$$\hat{H}_1 = e\boldsymbol{v}_0 \cdot \boldsymbol{A} \hat{a} \mathrm{e}^{-\mathrm{i}\omega t} + \mathrm{h.c.}, \tag{S12}$$

$$\hat{H}_2 = \frac{e^2}{2\gamma m_{\mathrm{e}}} \boldsymbol{A}^{\mathrm{T}} \boldsymbol{\Gamma} \boldsymbol{A} \hat{a}^2 \mathrm{e}^{-2\mathrm{i}\omega t} + \mathrm{h.c.}, \tag{S13}$$

$$\hat{H}_\phi = \frac{e^2}{\gamma m_{\mathrm{e}}} \boldsymbol{A}^\dagger \boldsymbol{\Gamma} \boldsymbol{A} \left(\hat{a}^\dagger \hat{a} + \frac{1}{2}\right). \tag{S14}$$

S2. The expression of scattering operator

The initial state of the combined free-electron-photon system, $|\Psi_{\rm i}\rangle = |\psi_{\rm e}\rangle \otimes |\psi_{\rm ph}\rangle$, satisfies

$$\mathrm{i}\hbar \frac{\partial}{\partial t}|\Psi_{\rm i}\rangle = \hat{H}_0|\Psi_{\rm i}\rangle,$$

where $\hat{H}_0$ is defined in Eq. (S7). To treat the interaction between free electrons and photons, it is convenient to work in the interaction picture. Eq. (S3) is already written in a form compatible with the interaction picture; only the electron free Hamiltonian needs to be included in the transformation. The interaction Hamiltonian in interaction picture is

$$\hat{V} = \mathrm{e}^{\frac{\mathrm{i}}{\hbar}\hat{H}_{\rm e}t}\hat{H}_{\rm int}(\boldsymbol{r},t)\mathrm{e}^{-\frac{\mathrm{i}}{\hbar}\hat{H}_{\rm e}t}. \tag{S15}$$

Because the transformation of position operator is $\mathrm{e}^{\frac{\mathrm{i}}{\hbar}\boldsymbol{v}_0\cdot\hat{\boldsymbol{p}}t}\boldsymbol{r}\mathrm{e}^{-\frac{\mathrm{i}}{\hbar}\boldsymbol{v}_0\cdot\hat{\boldsymbol{p}}t} = \boldsymbol{r} + \boldsymbol{v}_0 t$,substituting $\hat{H}_{\rm e}$ by the expression in Eq. (S8), the interaction Hamiltonian can be written as $\hat{V} = \hat{H}_{\rm int}(\boldsymbol{r} + \boldsymbol{v}_0 t, t)$. Since we assume the electron propagates along z-axis and only vector potential in interaction Hamiltonian is dependent on $\boldsymbol{r}$, this result is equivalent to applying the coordinate transformation

$$\hat{\boldsymbol{A}}(x,y,z,t) \to \hat{\boldsymbol{A}}(x,y,z+v_0 t,t).$$

Thus, in nonrecoil approximation, the final equivalent result is the vector potential in a co-moving-frame that assumes electrons keep initial velocities. The electrons coordinate can be expressed as $z' = z + v_0(t'-t)$, which can be rearranged as $t' = t - \left(\frac{z}{v_0} - \frac{z'}{v_0}\right)$ for light field. Substituting the retarded time, t', into the factor $\mathrm{e}^{-\mathrm{i}\omega t'}$ in Eq. (S12) and Eq. (S13), then we have

$$\mathrm{e}^{-\mathrm{i}\omega t'} = \mathrm{e}^{-\mathrm{i}\omega(t-(\frac{z}{v_0}-\frac{z'}{v_0}))} = \mathrm{e}^{-\mathrm{i}\omega\frac{z'}{v_0}}\mathrm{e}^{-\mathrm{i}\omega(t-\frac{z}{v_0})}.$$

Define electron energy-ladder operators

$$\hat{b} = \mathrm{e}^{\mathrm{i}\omega\left(t-\frac{z}{v_0}\right)}, \qquad \hat{b}^\dagger = \mathrm{e}^{-\mathrm{i}\omega\left(t-\frac{z}{v_0}\right)}, \tag{S16}$$

the factor can be recast as $\mathrm{e}^{-\mathrm{i}\omega t'} = \mathrm{e}^{-\mathrm{i}\omega\frac{z'}{v_0}}\hat{b}^\dagger$. Thus, we can write the interaction Hamiltonian as

$$\hat{V} = \hat{V}_1 + \hat{V}_2 + \hat{V}_\phi,$$

where

$$\hat{V}_1 = \sum_k \left(e\boldsymbol{v}_0\cdot\boldsymbol{A}\hat{a}\hat{b}^\dagger\mathrm{e}^{-\mathrm{i}\omega\frac{z'}{v_0}} + \mathrm{h.c.}\right)\hat{c}_k^\dagger\hat{c}_k, \tag{S17}$$

$$\hat{V}_2 = \sum_k \left(\frac{e^2}{2\gamma m_{\rm e}}\boldsymbol{A}^{\rm T}\boldsymbol{\Gamma}\boldsymbol{A}\hat{a}^2\hat{b}^{\dagger 2}\mathrm{e}^{-2\mathrm{i}\omega\frac{z'}{v_0}} + \mathrm{h.c.}\right)\hat{c}_k^\dagger\hat{c}_k, \tag{S18}$$

$$\hat{V}_\phi = \sum_k \left(\frac{e^2}{\gamma m_{\rm e}}\boldsymbol{A}^\dagger\boldsymbol{\Gamma}\boldsymbol{A}\left(\hat{a}^\dagger\hat{a} + \frac{1}{2}\right)\right)\hat{c}_k^\dagger\hat{c}_k. \tag{S19}$$

The evolution of system state from t_0 to t is governed by time-evolution operator $\hat{U}$, which can be written as $|\Psi(t)\rangle = \hat{U}(t,t_0)|\Psi(t_0)\rangle$ and $|\Psi(t_0 \to -\infty)\rangle = |\Psi_{\rm i}\rangle$. The time-evolution operator satisfies

$$\mathrm{i}\hbar\frac{\partial}{\partial t}\hat{U}(t,t_0) = \hat{V}\hat{U}(t,t_0).$$

Formally integrating yields the time-ordered exponential

$$\hat{U}(t,t_0) = \mathcal{T}\exp\left(-\frac{\mathrm{i}}{\hbar}\int_{t_0}^{t} dt'\,\hat{V}(z'(t'),t')\right).$$

Here, $\mathcal{T}$ is time-ordering operator. The time-integral can be transformed to coordinate-integral. Substituting dt' with $\frac{dz'}{v_0}$, the evolution operator as the function of coordinate can be expressed as

$$\hat{U}(z,z_0)=\mathcal{T}\exp\left(-\frac{\mathrm{i}}{\hbar}\int_{z_0}^{z}\frac{dz'}{v_0}\hat{V}(z')\right). \tag{S20}$$

Taking the expression of interaction Hamiltonian in Eq. (S17) into Eq. (S20), the integral of first-order interaction Hamiltonian $\hat{V}_1$ in the exponential is

$$-\frac{\mathrm{i}}{\hbar}\int_{z_0}^{z}\frac{dz'}{v_0}\hat{V}_1(z')=\sum_k\left(-g_{\mathrm{Qu}}^*\hat{b}^\dagger\hat{a}+g_{\mathrm{Qu}}\hat{b}\hat{a}^\dagger\right)\hat{c}_k^\dagger\hat{c}_k\,, \tag{S21}$$

where the inelastic quantum coupling constant is defined by

$$g_{\mathrm{Qu}}^*=\frac{e}{\hbar\omega}\int_{z_0}^{z}dz'\,E_z(z')\mathrm{e}^{-\mathrm{i}\omega\frac{z'}{v_0}}. \tag{S22}$$

Here, the relation of $\boldsymbol{A}(\boldsymbol{r})=-\mathrm{i}\frac{1}{\omega}\boldsymbol{E}(\boldsymbol{r})$ has been used.

Similarly, substituting $\hat{V}_2$ from Eq. (S18) into the integral, we obtain

$$-\frac{\mathrm{i}}{\hbar}\int_{z_0}^{z}\frac{dz'}{v_0}\hat{V}_2(z')=\sum_k\left(\frac{1}{2}g_2^*\hat{b}^{\dagger 2}\hat{a}^2-\frac{1}{2}g_2\hat{b}^2\hat{a}^{\dagger 2}\right)\hat{c}_k^\dagger\hat{c}_k\,, \tag{S23}$$

where the second-order of coupling constant is defined by

$$g_2^*=-\frac{\mathrm{i}}{\hbar}\frac{e^2}{p_0}\int_{z_0}^{z}dz'\,\boldsymbol{A}^{\mathrm{T}}(z')\boldsymbol{\Gamma}\boldsymbol{A}(z')\mathrm{e}^{-2\mathrm{i}\omega\frac{z'}{v_0}}.$$

The integral of $\hat{V}_\phi$ in the exponential is

$$-\frac{\mathrm{i}}{\hbar}\int_{z_0}^{z}\frac{dz'}{v_0}\hat{V}_\phi(z')=\sum_k\left(-\mathrm{i}g_\phi\left(\hat{a}^\dagger\hat{a}+\frac{1}{2}\right)\right)\hat{c}_k^\dagger\hat{c}_k\,. \tag{S24}$$

where the phase term is defined by

$$g_\phi=\frac{e^2}{\hbar p_0}\int_{z_0}^{z}dz'\,\boldsymbol{A}^\dagger(z')\boldsymbol{\Gamma}\boldsymbol{A}(z').$$

Taking the integral of the Hamiltonian interaction term in Eq. (S21), (S23) and (S24), into Eq. (S20), the expression of evolution operator is

$$\hat{U}(z,z_0)=\mathcal{T}\exp\sum_k\left(g_{\mathrm{Qu}}\hat{b}\hat{a}^\dagger-g_{\mathrm{Qu}}^*\hat{b}^\dagger\hat{a}+\frac{1}{2}g_2^*\hat{b}^{\dagger 2}\hat{a}^2-\frac{1}{2}g_2\hat{b}^2\hat{a}^{\dagger 2}-\mathrm{i}g_\phi\left(\hat{a}^\dagger\hat{a}+\frac{1}{2}\right)\right)\hat{c}_k^\dagger\hat{c}_k.$$

Since the temporal integral has been transformed into a spatial integral in Eq. S20, the evolution operator does not obviously show time-dependence. However, g_{Qu}, g_2, g_ϕ and their conjugates are time-dependent. The time-ordering operator is applied to those coupling parameters. Extend the integral to infinity, we have the scattering operator $\hat{S}=\hat{U}(z\to\infty,z_0\to-\infty)$.

Furthermore, according to Zassenhaus formula (*78*), which can be expressed as $\mathrm{e}^{t(X+Y)}=\mathrm{e}^{tX}\mathrm{e}^{tY}\mathrm{e}^{-\frac{t^2}{2}[X,Y]}\mathrm{e}^{\frac{t^3}{6}(2[Y,[X,Y]]+[X,[X,Y]])}\cdots$, scattering operator can be separated into a product of infinite factors and each factor only includes g_{Qu}, g_2 or g_ϕ. In the first-order approximation, the scattering operator only includes the displacement operator $\hat{D}(g_{\mathrm{Qu}}\hat{b})=\mathrm{e}^{\sum_k(g_{\mathrm{Qu}}\hat{b}\hat{a}^\dagger-g_{\mathrm{Qu}}^*\hat{b}^\dagger\hat{a})\hat{c}_k^\dagger\hat{c}_k}$, the squeeze operator $\hat{\mathcal{S}}(g_2\hat{b}^2)=\mathrm{e}^{\sum_k\left(\frac{1}{2}g_2^*\hat{b}^{\dagger 2}\hat{a}^2-\frac{1}{2}g_2\hat{b}^2\hat{a}^{\dagger 2}\right)\hat{c}_k^\dagger\hat{c}_k}$ and the photon number operator $\hat{N}=\hat{a}^\dagger a$, that is

$$\hat{S}\cong\mathcal{T}\hat{D}(g_{\mathrm{Qu}}\hat{b})\hat{\mathcal{S}}(g_2\hat{b}^2)\mathrm{e}^{-\mathrm{i}\sum_k g_\phi\left(\hat{N}+\frac{1}{2}\right)\hat{c}_k^\dagger\hat{c}_k}.$$

The higher order terms include the product of $g_{\mathrm{Qu}}g_2$, $g_{\mathrm{Qu}}g_\phi$, $g_2 g_\phi$ and its complex conjugate. Generally, $g_{\mathrm{Qu}} < 1$, g_2 and $g_\phi \ll 1$, thus, we can safely neglect the higher order terms.

S3. The phase dependent on local density of states

Using Magnus expansion (*79*), the expression of evolution operator can be written without an explicit time-ordering operator as $\hat{U}(t,t_0) = \exp(\sum_{l=1}^{\infty}\Omega_l)$. The quantities Ω_1, Ω_2, and higher-order terms are successive Magnus terms obtained from the interaction Hamiltonian and its commutators,

$$\Omega_1 = -\frac{\mathrm{i}}{\hbar}\int_{t_0}^{t} dt_1\,\hat{V}(t_1),$$

$$\Omega_2 = \frac{1}{2}\left(-\frac{\mathrm{i}}{\hbar}\right)^2 \int_{t_0}^{t} dt_1 \int_{t_0}^{t_1} dt_2\,[\hat{V}(t_1),\hat{V}(t_2)] \qquad \text{(S25)}$$

$$\cdots$$

If we retain only the first-order term, we recover Eq. (S24) without the time-ordering operator. The second-order term depends on the commutator of interaction Hamiltonians at different times,

$$[\hat{V}(t_1),\hat{V}(t_2)] = [\hat{V}_1(t_1),\hat{V}_1(t_2)] + [\hat{V}_1(t_1),\hat{V}_2(t_2)] + [\hat{V}_2(t_1),\hat{V}_1(t_2)] + \cdots$$

Neglect all high order terms and only consider the commutator of first-order interaction Hamiltonian, then we have

$$[\hat{V}(t_1),\hat{V}(t_2)] \approx [\hat{V}_1(t_1),\hat{V}_1(t_2)].$$

The electron energy-ladder operators in Eq. (S16) can be expressed as

$$\hat{b} = \textstyle\sum_K \hat{c}_{K-q}^{\dagger}\hat{c}_K,\ \ \hat{b}^{\dagger} = \textstyle\sum_K \hat{c}_{K+q}^{\dagger}\hat{c}_K.$$

To simplify the calculation, mark $\frac{z'}{v_0}$ as t and let $e\boldsymbol{v}_0\cdot\boldsymbol{A}\mathrm{e}^{-\mathrm{i}\omega t} = h(t)$, Eq. (S17) can be recast as

$$\hat{V}_1 = \sum_k h(t)\hat{a}\sum_K \hat{c}_{K+q}^{\dagger}\hat{c}_K\,\hat{c}_k^{\dagger}\hat{c}_k + \mathrm{h.c.}$$

The application of the fermion operator product, $\hat{c}_{K+q}^{\dagger}\hat{c}_K\hat{c}_k^{\dagger}\hat{c}_k$, to an electron state with momentum k is not equal to zero only when $K = k$. Thus, we have

$$\hat{V}_1 = h(t)\hat{a}\sum_k \hat{c}_{k+q}^{\dagger}\hat{c}_k + \mathrm{h.c.}$$

Then

$$[\hat{V}_1(t_1),\hat{V}_1(t_2)] = \sum_{k,k'}[h(t_1)\hat{a}\hat{c}_{k+q}^{\dagger}\hat{c}_k + h^*(t_1)\hat{a}^{\dagger}\hat{c}_{k-q}^{\dagger}\hat{c}_k\,, h(t_2)\hat{a}\hat{c}_{k'+q}^{\dagger}\hat{c}_{k'} + h^*(t_2)\hat{a}^{\dagger}\hat{c}_{k'-q}^{\dagger}\hat{c}_{k'}]$$

$$= \textstyle\sum_{k,k'}\left\{\underbrace{h(t_1)h(t_2)\hat{a}^2[\hat{c}_{k+q}^{\dagger}\hat{c}_k,\ \hat{c}_{k'+q}^{\dagger}\hat{c}_{k'}]}_{I} + \underbrace{h^*(t_1)h^*(t_2)\,\hat{a}^{\dagger 2}\,[\hat{c}_{k-q}^{\dagger}\hat{c}_k,\ \hat{c}_{k'-q}^{\dagger}\hat{c}_{k'}]}_{II} + \right.$$

$$\left.\underbrace{h(t_1)h^*(t_2)[\hat{a}\hat{c}_{k+q}^{\dagger}\hat{c}_k,\ \hat{a}^{\dagger}\hat{c}_{k'-q}^{\dagger}\hat{c}_{k'}]}_{III} + \underbrace{h^*(t_1)h(t_2)[\hat{a}^{\dagger}\hat{c}_{k-q}^{\dagger}\hat{c}_k,\ \hat{a}\hat{c}_{k'+q}^{\dagger}\hat{c}_{k'}]}_{IV}\right\}.$$

The commutation relation in *I* and *II* nullifies, $[\hat{c}_{k+q}^{\dagger}\hat{c}_k,\ \hat{c}_{k'+q}^{\dagger}\hat{c}_{k'}] = [\hat{c}_{k-q}^{\dagger}\hat{c}_k,\ \hat{c}_{k'-q}^{\dagger}\hat{c}_{k'}] = 0$. The commutation in *III* is,

$$[\hat{a}\hat{c}_{k+q}^{\dagger}\hat{c}_k,\hat{a}^{\dagger}\hat{c}_{k'-q}^{\dagger}\hat{c}_{k'}] = \hat{a}\hat{a}^{\dagger}[\hat{c}_{k+q}^{\dagger}\hat{c}_k,\hat{c}_{k'-q}^{\dagger}\hat{c}_{k'}] + [\hat{a},\hat{a}^{\dagger}]\hat{c}_{k'-q}^{\dagger}\hat{c}_{k'}\hat{c}_{k+q}^{\dagger}\hat{c}_k$$

$$= 0 + \hat{c}^\dagger_{k'-q}\hat{c}_{k'}\hat{c}^\dagger_{k+q}\hat{c}_k = \hat{c}^\dagger_{k'-q}\hat{c}_k - \hat{c}^\dagger_{k'-q}\hat{c}^\dagger_{k+q}\hat{c}_{k'}\hat{c}_k.$$

$\hat{c}^\dagger_{k'-q}\hat{c}^\dagger_{k+q}\hat{c}_{k'}\hat{c}_k$ is always equal to zero and only if $k' = k + q$, $\hat{c}^\dagger_{k'-q}\hat{c}_k$ not always zero.
Thus, part *III* is

$$(III)\colon \sum_{k,k'} h(t_1)h^*(t_2)\left[\hat{a}\hat{c}^\dagger_{k+q}\hat{c}_k,\ \hat{a}^\dagger\hat{c}^\dagger_{k'-q}\hat{c}_{k'}\right] = \sum_k h(t_1)h^*(t_2)\,\hat{c}^\dagger_k\hat{c}_k.$$

Similarly, the commutation for *IV* is

$$\left[\hat{a}^\dagger\hat{c}^\dagger_{k-q}\hat{c}_k, \hat{a}\hat{c}^\dagger_{k'+q}\hat{c}_{k'}\right] = \hat{a}^\dagger\hat{a}\left[\hat{c}^\dagger_{k-q}\hat{c}_k, \hat{c}^\dagger_{k'+q}\hat{c}_{k'}\right] + [\hat{a}^\dagger, \hat{a}]\hat{c}^\dagger_{k'+q}\hat{c}_{k'}\hat{c}^\dagger_{k-q}\hat{c}_k$$
$$= 0 - \hat{c}^\dagger_{k'+q}\hat{c}_{k'}\hat{c}^\dagger_{k-q}\hat{c}_k = -\hat{c}^\dagger_{k'+q}\hat{c}_k + \hat{c}^\dagger_{k'+q}\hat{c}^\dagger_{k-q}\hat{c}_{k'}\hat{c}_k,$$

and part *IV* is

$$(IV)\colon \sum_{k,k'} h^*(t_1)h(t_2)\left[\hat{a}^\dagger\hat{c}^\dagger_{k-q}\hat{c}_k,\ \hat{a}\hat{c}^\dagger_{k'+q}\hat{c}_{k'}\right] = -\sum_k h(t_1)h^*(t_2)\,\hat{c}^\dagger_k\hat{c}_k.$$

Substituting *III* and *IV* to the term $\left[\hat{V}_1(t_1), \hat{V}_1(t_2)\right]$ results in

$$\left[\hat{V}_1(t_1), \hat{V}_1(t_2)\right] = \sum_k\left(h(t_1)h^*(t_2)\hat{c}^\dagger_k\hat{c}_k - h^*(t_1)h(t_2)\hat{c}^\dagger_k\hat{c}_k\right) = \sum_k \mathrm{Im}\{h(t_1)h^*(t_2)\}\hat{c}^\dagger_k\hat{c}_k.$$

We now turn to resolve the term $\mathrm{Im}\{h(t_1)h^*(t_2)\}$ and write Ω_2 of Eq. (S25) explicitly.
$h(t)$ is given by $h(t) = e\boldsymbol{v}_0 \cdot \boldsymbol{A}\mathrm{e}^{-\mathrm{i}\omega t}$. Substituting it into Eq. (S25), we have the second order of Magnus expansion

$$\Omega_2(t, t_0) \approx -\mathrm{i}\frac{1}{\hbar^2}\sum_k \hat{c}^\dagger_k\hat{c}_k \int_{t_0}^{t} dt_1 \int_{t_0}^{t_1} dt_2\, \mathrm{Im}\left\{\left(e\boldsymbol{v_0}\cdot\boldsymbol{A}(t_1)\mathrm{e}^{-\mathrm{i}\omega t_1}\right)\left(e\boldsymbol{v_0}\cdot\boldsymbol{A}^*(t_2)\right)\mathrm{e}^{\mathrm{i}\omega t_2}\right\}.$$

Transfer the temporal integral to spatial integral, along the electron propagation axis, z,

$$\Omega_2(z, z_0) \approx -\mathrm{i}\left(\frac{e}{\hbar\omega}\right)^2 \sum_k \hat{c}^\dagger_k\hat{c}_k \int_{z_0}^{z} dz_1 \int_{z_0}^{z_1} dz_2\, \mathrm{Im}\left\{E_z(z_1)\mathrm{e}^{-\mathrm{i}\omega\frac{z_1}{v_0}} E_z^*(z_2)\mathrm{e}^{\mathrm{i}\omega\frac{z_2}{v_0}}\right\}.$$

Define $\Omega_2 = \mathrm{i}\sum_k \hat{c}^\dagger_k\hat{c}_k\,\chi_\omega$. The quantity χ_ω is a purely phase-like term. Extending the integration limits to infinity, we obtain

$$\chi_\omega = -\mathrm{Im}\left\{\int_{-\infty}^{+\infty} dz\, g^*_{\mathrm{Qu}}(z)\frac{d}{dz}g_{\mathrm{Qu}}(z)\right\}. \tag{S26}$$

Here, Eq. (S22) has been used. This result indicates that χ_ω is the integral over a closed loop in the complex g_{Qu}-plane. It can be rewritten as a compact form: $\chi_\omega = -\mathrm{Im}\{\oint g^*_{\mathrm{Qu}} dg_{\mathrm{Qu}}\}$. The expression in Eq. (S26) demonstrates that χ_ω has the same form of geometric phase (*80*).
Photon annihilation or creation operators do not appear in Eq. (S26), thus, χ_ω is independent of light intensity. Accounting for all optical modes, the total phase is $\chi = \int_0^{+\infty} d\omega\,\chi_\omega$.
Finally, the scattering operator can be written as

$$\hat{S} = \exp\left(\mathrm{i}\sum_k \hat{c}^\dagger_k\hat{c}_k\,\chi\right)\exp\sum_k \hat{c}^\dagger_k\hat{c}_k\left(g_{\mathrm{Qu}}\hat{b}\hat{a}^\dagger - g^*_{\mathrm{Qu}}\hat{b}^\dagger\hat{a} + \frac{1}{2}g_2^*\hat{b}^{\dagger 2}\hat{a}^2 - \frac{1}{2}g_2\hat{b}^2\hat{a}^{\dagger 2} - \mathrm{i}g_\phi\left(\hat{a}^\dagger\hat{a} + \frac{1}{2}\right)\right),$$

the same as the expression shown in Eq. (3) in main text.

S4. Static analysis of optical microresonator

Fig. S1 shows a single optical all-pass microresonator. The basic relation among the incident field $E_1\mathrm{e}^{i\omega t}$ (the incident power $P_{\mathrm{inc}} \propto |E_1\mathrm{e}^{i\omega t}|^2$), the output field $E_2\mathrm{e}^{i\omega t}$ (the output power $P_{\mathrm{out}} \propto |E_2\mathrm{e}^{i\omega t}|^2$), the circulating field just before the coupling point $E_3\mathrm{e}^{i\omega t}$ (the circulating power $P_{\mathrm{cir}} \propto |E_3\mathrm{e}^{i\omega t}|^2$) and after the coupling point $E_4\mathrm{e}^{i\omega t}$ are related by the following unitary matrix (*74*):

$$\begin{pmatrix} E_2 \\ E_4 \end{pmatrix} = \begin{pmatrix} r & \mathrm{i}\kappa \\ \mathrm{i}\kappa & r \end{pmatrix}\begin{pmatrix} E_1 \\ E_3 \end{pmatrix} \tag{S27}$$

where the lumped self- and cross-coupling coefficients r and κ satisfy the relation $r^2 + \kappa^2 = 1$, both r and κ are real numbers. In steady state, the circulating fields E_3 and E_4 are connected by round-trip relation: $E_3 = a\mathrm{e}^{\mathrm{i}\phi_\mathrm{R}} E_4$. Here, $\phi_\mathrm{R} = \omega T_\mathrm{R}$, T_R is the round-trip time of light in resonator. a represents the single-pass amplitude transmission and $0 < a < 1$. From the matrix in Eq. (S27), we can derive the relation in steady state:

$$E_{4,\mathrm{s}} = \frac{i\kappa}{1-x} E_1,\ E_{2,\mathrm{s}} = \frac{r - a\mathrm{e}^{\mathrm{i}\phi_\mathrm{R}}}{1-x} E_1,\ E_{3,\mathrm{s}} = a\mathrm{e}^{\mathrm{i}\phi_\mathrm{R}} E_{4,\mathrm{s}},$$

where $x = ra\mathrm{e}^{\mathrm{i}\phi_\mathrm{R}}$ is the one round trip factor.

The ratio of circulating power to incident power, or the buildup factor B, can be expressed as the function of normalized detuning

$$B(\phi_\mathrm{R}) = \frac{P_\mathrm{cir}(\phi_\mathrm{R})}{P_\mathrm{inc}} = \left|\frac{E_{3,\mathrm{s}}}{E_1}\right|^2 = \frac{(1-r^2)a^2}{1 - 2ra\cos\phi_\mathrm{R} + r^2a^2}.$$

At the resonant point ($\phi_\mathrm{R} = m2\pi, m$ is an integer), the buildup factor can be reduced to $B_\mathrm{res} = \frac{(1-r^2)a^2}{(1-ra)^2}$. Furthermore, the non-resonant and resonant buildup factor is related by $B(\phi_\mathrm{R}) = B_\mathrm{res}\frac{1}{1+b(1-\cos\phi_\mathrm{R})}$. Here, the parameter $b = \frac{2ra}{(1-ra)^2}$. If the attenuation is negligible ($a = 1$) and self-coupling coefficient is near to unit, then $b \xrightarrow[a\to 1, r\to 1]{} \frac{1}{2} B_\mathrm{res}^2$. The first-order derivation of buildup factor is

$$\frac{d}{d\phi_\mathrm{R}} B(\phi_\mathrm{R}) = \frac{-(1-r^2)a^2 2ra\sin\phi_\mathrm{R}}{(1 - 2ra\cos\phi_\mathrm{R} + r^2a^2)^2}. \tag{S28}$$

To evaluate the sensitivity near the edge, we examine the half-maximum of the resonance fringe, where $B(\phi_0) = \frac{1}{2} B_\mathrm{res}$. This occurs at

$$\phi_0 = \arccos(1 - 1/b). \tag{S29}$$

In fact, ϕ_0 is close to the point that most sensitive to detuning. At the half-maximum of the resonance fringe, the change of buildup factor due to a small phase shift is

$$B(\phi_0) - B(\phi_0 + \delta\phi) \cong \frac{\sqrt{2}}{4} B_\mathrm{res}\sqrt{b}\,\delta\phi \xrightarrow[a\to 1, r\to 1]{} \frac{1}{4} B_\mathrm{res}^2 \delta\phi = \frac{1}{\pi^2}\mathcal{F}^2\delta\phi,$$

where $\mathcal{F}$ is finesse, related to buildup factor with relation $\mathcal{F} = \frac{\pi}{2} B_\mathrm{res}$ in an all-pass resonator (*12*).

S5. Dynamic analysis of optical microresonator

For an all-pass resonator, we assume that the input field is quasi-monochromatic and has constant amplitude. A time-dependent phase shift $\delta\phi(t)$ inside the cavity produces corresponding variations in the circulating and output fields. We further assume that the phase shift amplitude is small, satisfied $|\delta\phi(t)| \ll 1$.

The time-dependent circulating field $E_4(t)$ evolves: 1. Current input contribution due to part of $E_1(t)$ couples into the resonator via κ. 2. Previous circulation contribution due to the field $E_4(t - T_\mathrm{R})$ from one round-trip earlier, attenuated by a and phase-shift by $\phi_\mathrm{R} + \delta\phi(t)$. Combining the field relations and round-trip dynamics: $E_4(t) = \mathrm{i}\kappa E_1(t) + rE_3(t)$ and $E_3(t) = a\mathrm{e}^{\mathrm{i}(\phi_\mathrm{R}+\delta\phi(t))} E_4(t - T_\mathrm{R})$ (before the perturbation, $E_3(t) = a\mathrm{e}^{\mathrm{i}\phi_\mathrm{R}} E_4(t - T_\mathrm{R})$), we have the time-domain recursion:

$$E_4(t) = \mathrm{i}\kappa E_1 + x\mathrm{e}^{\mathrm{i}\delta\phi(t)} E_4(t - T_\mathrm{R}).$$

Separating the $E_4(t)$ to steady-state part and perturbation part,

$$E_4(t) = E_{4,\mathrm{s}} + \delta E_4(t), \tag{S30}$$

where $\delta E_4(t)$ is the small deviation due to $\delta\phi(t)$, thus

$$\underbrace{E_{4,\mathrm{s}} + \delta E_4(t)}_{E_4(t)} = \mathrm{i}\kappa E_1 + x\mathrm{e}^{\mathrm{i}\delta\phi(t)}\underbrace{\left(E_{4,\mathrm{s}} + \delta E_4(t - T_\mathrm{R})\right)}_{E_4(t-T_\mathrm{R})}.$$

Because $E_{4,\mathrm{s}} = \mathrm{i}\kappa E_1 + xE_{4,\mathrm{s}}$, after canceling the common $E_{4,\mathrm{s}}$ on both sides, we get the recursion

$$\delta E_4(t) = xe^{i\delta\phi(t)}\delta E_4(t - T_\mathrm{R}) + \left(e^{i\delta\phi(t)} - 1\right)xE_{4,\mathrm{s}}.$$

The solution of this closed-form series can be found by unfolding that recurrence for t after the perturbation starts,

$$\delta E_4(t - T_\mathrm{R}) = xe^{i\delta\phi(t-T_\mathrm{R})}\delta E_4(t - 2T_\mathrm{R}) + \left(e^{i\delta\phi(t-T_\mathrm{R})} - 1\right)xE_{4,\mathrm{s}}.$$

$$\delta E_4(t - 2T_\mathrm{R}) = xe^{i\delta\phi(t-2T_\mathrm{R})}\delta E_4(t - 3T_\mathrm{R}) + \left(e^{i\delta\phi(t-2T_\mathrm{R})} - 1\right)xE_{4,\mathrm{s}}.$$

$$\cdots$$

Combining all recurrence relationships, the exact solution is

$$\delta E_4(t) = E_{4,\mathrm{s}}\sum_{n=0}^{\infty} x^{n+1}\cdot\left(e^{i\sum_{m=0}^{n-1}\delta\phi(t-mT_\mathrm{R})}\right)\left(\mathrm{e}^{\mathrm{i}\delta\phi(t-nT_\mathrm{R})} - 1\right). \tag{S31}$$

Since $\sum_{m=0}^{n-1}\delta\phi(t - mT_\mathrm{R})$ is small, neglecting this term, the perturbation of the circulating field can be reduced to

$$\delta E_4(t) = E_{4,\mathrm{s}}\sum_{n=0}^{\infty} x^{n+1}\cdot\left(\mathrm{e}^{\mathrm{i}\delta\phi(t-nT_\mathrm{R})} - 1\right).$$

Hence, combining Eq. (S30) and (S31), we have the total circulating field.

Similarly, the output field $E_2(t)$ evolves: 1. Current input contribution due to part of $E_1(t)$ output via reflection. 2. Circulation contribution due to the field $E_3(t)$ couples into the resonator via κ. Combining the field relations and round-trip dynamics: $E_2(t) = rE_1(t) + \mathrm{i}\kappa E_3(t)$ and $E_3(t) = a\mathrm{e}^{\mathrm{i}(\phi_\mathrm{R}+\delta\phi(t))}E_4(t - T_\mathrm{R})$. The output fields are

$$E_2(t) = rE_1(t) + \mathrm{i}\kappa a\mathrm{e}^{\mathrm{i}(\phi_\mathrm{R}+\delta\phi(t))}E_4(t - T_\mathrm{R}).$$

Replace $E_2(t) = E_{2,\mathrm{s}} + \delta E_2(t)$, we have

$$E_{2,\mathrm{s}} + \delta E_2(t) = rE_1(t) + \mathrm{i}\kappa a\mathrm{e}^{\mathrm{i}\phi_\mathrm{R}}\left(E_{4,\mathrm{s}} + \delta E_4(t - T_\mathrm{R})\right) + \mathrm{i}\kappa a\mathrm{e}^{\mathrm{i}\phi_\mathrm{R}}\left(\mathrm{e}^{\mathrm{i}\delta\phi(t)} - 1\right)E_4(t - T_\mathrm{R}).$$

Because $E_{2,\mathrm{s}} = rE_1 + \mathrm{i}\kappa a\mathrm{e}^{\mathrm{i}\phi_\mathrm{R}}E_{4,\mathrm{s}}$, after canceling the common terms on both sides, we get $\delta E_2(t) = \mathrm{i}\kappa a\mathrm{e}^{\mathrm{i}\phi_\mathrm{R}}\delta E_4(t - T_\mathrm{R}) + \mathrm{i}\kappa a\mathrm{e}^{\mathrm{i}\phi_\mathrm{R}}\left(\mathrm{e}^{\mathrm{i}\delta\phi(t)} - 1\right)E_{4,\mathrm{s}} = \mathrm{i}\frac{\kappa}{r}\delta E_4(t)$. Thus, the transient response of output field is

$$E_2(t) = E_{2,\mathrm{s}} + \mathrm{i}\frac{\kappa}{r}\delta E_4(t). \tag{S32}$$

S6. The net energy output at the side of the resonance fringe

In the all-pass microresonator, assuming the input power is in steady state, the time-dependent phase-shift on the circulating field leads to a time-dependent output power $P_\mathrm{out}(t) = P_\mathrm{inc}\left|\frac{E_2(t)}{E_1}\right|^2$ and therefore to a net output energy

$$\mathcal{E}_\mathrm{net} = \int dt\left(P_\mathrm{out}(t) - P_\mathrm{out,s}\right), \tag{S33}$$

where $P_\mathrm{out,s} = P_\mathrm{inc}\left|\frac{E_{2,\mathrm{s}}}{E_1}\right|^2$ is the steady state power output.

If the resonator is locked at the side of the resonant fringe, as shown in Fig. 3b, then substituting Eq. (S31) and (S32) into the expression of $P_{\text{out}}(t)$ gives

$$P_{\text{out}}(t) \approx P_{\text{out,s}} + P_{\text{inc}} \frac{\kappa^2}{|1-x|^2} 2\text{Re}\left[\left(\frac{1}{r^2}x^* - 1\right)\frac{\delta E_4(t)}{E_{4,\text{s}}}\right].$$

Net energy output due to the small phase shift in the cavity can be calculated by

$$\mathcal{E}_{\text{net}} \approx 2P_{\text{inc}} \frac{\kappa^2}{|1-x|^2} \int_{-\infty}^{+\infty} dt \, \text{Re}\left[\left(\frac{1}{r^2}x^* - 1\right)\frac{\delta E_4(t)}{E_{4,\text{s}}}\right]. \quad \text{(S34)}$$

Assuming the phase shift lasts from $t = 0$ to $t = T_{\text{int}}$ and the phase shift amplitude is a constant,

$$\delta\phi(t) = \begin{cases} 0 & \text{for } t < 0 \text{ and } t \geq T_{\text{int}} \\ \delta\phi & \text{for } 0 \leq t < T_{\text{int}} \end{cases}.$$

If the duration of the phase shifts is no more than one round trip time, i.e., $T_{\text{int}} \leq T_{\text{R}}$, then

$$\frac{\delta E_4(t)}{E_{4,\text{s}}} = \begin{cases} 0 & \text{for } t < T_{\text{R}} \\ x^n\left(e^{i\delta\phi} - 1\right) & \text{for } nT_{\text{R}} \leq t < nT_{\text{R}} + T_{\text{int}} \\ 0 & \text{for } nT_{\text{R}} + T_{\text{int}} \leq t < (n+1)T_{\text{R}} \end{cases},$$

where $n = \lceil t/T_{\text{R}} \rceil$. The integral of real part in Eq. (S32) can be transformed into

$$\begin{aligned}
&\int_{-\infty}^{+\infty} dt \, \text{Re}\left[\left(\frac{1}{r^2}x^* - 1\right)\frac{\delta E_4(t)}{E_{4,\text{s}}}\right]. \\
&= \sum_{n=1}^{\infty} \int_{nT_{\text{R}}}^{(n+1)T_{\text{R}}} dt \, \text{Re}\left[\left(\frac{1}{r^2}x^* - 1\right) x^n\left(e^{i\delta\phi} - 1\right)\right]. \\
&= \sum_{n=1}^{\infty} T_{\text{int}} \text{Re}\left[\left(\frac{1}{r^2}x^* - 1\right) x^n\left(e^{i\delta\phi} - 1\right)\right]. \\
&= T_{\text{int}} \text{Re}\left[\left(e^{i\delta\phi} - 1\right)\frac{a^2 - x}{1 - x}\right]. \\
&\approx \frac{T_{\text{int}}\delta\phi}{|1-x|^2} ra(1 - a^2)\sin\phi_{\text{R}}.
\end{aligned}$$

Substituting this result into Eq. (S34), we have

$$\mathcal{E}_{\text{net}} \approx P_{\text{inc}} 2T_{\text{int}}\delta\phi \frac{ra(1-r^2)(1-a^2)\sin\phi_{\text{R}}}{(1-2ra\cos\phi_{\text{R}} + r^2a^2)^2}.$$

Here, some factors can be replaced by the first-order derivation of intra-cavity buildup factor shown in Eq. (S28). Thus, the net energy output can be simplified to

$$\mathcal{E}_{\text{net}} = -P_{\text{inc}} T_{\text{int}} \delta\phi \frac{dB(\phi_{\text{R}})}{d\phi_{\text{R}}} (1 - a^2) \frac{1}{a^2}. \quad \text{(S35)}$$

This expression shows that net output energy depends on two factors: 1. The phase sensitivity of intracavity buildup factor $\frac{dB(\phi_{\text{R}})}{d\phi_{\text{R}}}$. 2. The intensity loss of every round trip $(1 - a^2)$. For lossless resonator, $\mathcal{E}_{\text{net}} = 0$, it is impossible to detect net energy output.

If the detuning phase is set at half-max point, $\phi_{\text{R}} = \phi_0$, then

$$\mathcal{E}_{\text{net}} = -P_{\text{inc}} T_{\text{int}} \delta\phi \frac{(1-r^2)(1-a^2)\sqrt{ra}}{2(1-ra)^3}.$$

The finesse of all-pass resonator can be express as $\mathcal{F} = \frac{\text{FSR}_{\phi_{\text{R}}}}{\text{FWHM}_{\phi_{\text{R}}}} = \frac{\pi\sqrt{ra}}{1-ra}$. The circulating light intensity $I_{\text{cir}} = I_{\text{inc}} \frac{2\mathcal{F}}{\pi}$. For high-finesse microresonator $(a \to 1, r \to 1)$, $\frac{(1-r^2)(1-a^2)}{(1-ra)^2} \approx 2$. Taking those expressions to above equation, then we have

$$\mathcal{E}_{\text{net}} \xrightarrow[a\to 1, r\to 1]{} -\frac{1}{2} P_{\text{cir}} T_{\text{int}} \delta\phi.$$

Thus, the output energy is proportional to the circulating light intensity.

S7. Signal-to-noise ratio analysis

In the all-pass microresonator, the single electron-induced additional outgoing photon number is $\delta N = \mathcal{E}_{\mathrm{net}}/(N_{\mathrm{e}}\hbar\omega)$, the net energy output $\mathcal{E}_{\mathrm{net}}$ is defined in Eq. (S33). Assuming the dominant noise source is the quantum fluctuation of coherent light, which scales as the square root of photon number in the output port, $\Delta N = \sqrt{\overline{N}}$. As shown in Fig. 3c in the manuscript, the noise from steady state power output nullifies for critical coupling, $\phi_{\mathrm{R}} = 0$.

After the electron passage, the duration of the signal in the resonator is approximately equal to the cavity lifetime. The signal-to-noise ratio (SNR) can be written as the additional

$$\mathrm{SNR} = \frac{\delta N}{\Delta N} = \frac{\mathcal{E}_{\mathrm{net}}/(N_{\mathrm{e}}\hbar\omega)}{\sqrt{P_{\mathrm{out}}T_{\mathrm{C}}/(\hbar\omega)}}. \tag{S36}$$

Here, $P_{\mathrm{out}}T_{\mathrm{C}}$ is the total energy output, where T_{C} is the cavity lifetime, can be expressed as $T_{\mathrm{C}} = Q/\omega$, Q is the quality factor. Fig. S3 shows the ϕ_{R}-dependent SNR. To understand the signal in Fig. S3, note that for an output field comprising the unperturbed pump component, $E_{2,\mathrm{s}}$, and the part influenced by the electron, δE_2, as shown in Eq. (S32). The power output would be proportional to $|E_{2,\mathrm{s}}|^2 + 2\mathrm{Re}(E_{2,\mathrm{s}}\delta E_2) + |\delta E_2|^2$. Thus, for pumping on resonance $\phi_{\mathrm{R}} = 0$, $E_{2,\mathrm{s}} = 0$ and the noise is purely emanating from the signal field. However, for the steepest slope of the spectral line shape the signal is higher, $2\mathrm{Re}(E_{2,\mathrm{s}}\delta E_2)$, and so is the noise, which is proportional to $|E_{2,\mathrm{s}}|$. In Fig. S3 we show total photon number output, proportional to $|E_{2,s} + \delta E_2|^2$, and net photon number output, proportional to $2\mathrm{Re}(E_{2,\mathrm{s}}\delta E_2) + |\delta E_2|^2$, which reaches 500 photons in the interference term and the corresponding SNR ≈ 0.6. However, this is not the only way to detect the output. The optimum should be chosen with respect to the experimental system. For example, pumping on the cavity resonance, $\phi_{\mathrm{R}} = 0$, allows for a dark output port, without the noise of the pump field. In the example here, every electron would yield approximately 0.8 photon (as the mean of a coherent state), detectable either directly, or via homodyne detection with a reference field.

S8. The electron number variance

This section exemplifies how the measurement of light that was scattered elastically from passing electrons can improve the statistical variance of the electron state, enabling sub-Poisson (i.e., quantum) distribution. Assuming that the initial electron distribution is Poissonian, the probability for an exact number of electrons m is $P(m) = e^{-\overline{N_{\mathrm{e}}}}\frac{\overline{N_{\mathrm{e}}}^m}{m!}$, where $\overline{N_{\mathrm{e}}}$ is the mean electron number. The variance is $\overline{N_{\mathrm{e}}}$ and the standard-deviation fluctuation is $\sqrt{\overline{N_{\mathrm{e}}}}$. In the output port of the resonator, the expectation value for the photon number ($\overline{N}$) estimated over the cavity lifetime (T_{C}), depends on the electron number (N_{e}) through the output power (P_{out}), as $\overline{N} = \frac{P_{\mathrm{out}}T_{\mathrm{C}}}{\hbar\omega}$. We write $P_{\mathrm{out}}T_{\mathrm{C}} = P_{\mathrm{out,s}}T_{\mathrm{C}} + \mathcal{E}_{\mathrm{net}}(\delta\phi)$, given that $P_{\mathrm{out,s}}$ as the output power in a steady state, without passing electrons, and an additional energy due to the electrons is $\mathcal{E}_{\mathrm{net}}(\delta\phi)$, where $\delta\phi = -g_{\phi}N_{\mathrm{e}}$. Thus, $\overline{N}$ is the function of the electron number, $\overline{N} = \overline{N}(N_{\mathrm{e}})$. For an exact electron number $N_{\mathrm{e}} = m$, the probability of find l photons in output port is $P(l|m) = e^{-\overline{N}(m)}\frac{(\overline{N}(m))^l}{l!}$.

Using Bayes' theorem, we can estimate the electron number, m, given a measured photon number, l, as

$$P(m|l) = \frac{P(l|m) \cdot P(m)}{P(l)}.$$

The probability to find exactly l photons is $P(l) = \sum_m P(l|m) \cdot P(m)$. The posterior expected electron number is $\langle N_e \rangle = \sum_m m \cdot P(m|l)$, with a standard deviation of $\Delta N_e = \sqrt{\sum_m (m - \langle N_e \rangle)^2 P(m|l)}$.

Since there is no general analytical form for ΔN_e, we turn to consider the example discussed in the main text, where the pump is detuned to the side of the resonance fringe. By combining Eq. (8) and (13) in the main text, the net energy output can be expressed as $\mathcal{E}_{net} \approx \frac{1}{2} P_{cir} T_{int} g_\phi \overline{N_e}$, and the expected photon number is $\overline{N} = \frac{1}{\hbar\omega}\left(P_{out,s} T_C + \frac{1}{2} P_{cir} T_{int} g_\phi \overline{N_e}\right)$. For an intense pump, $\overline{N} \gg 1$, we approximate $P(l|m) \approx \frac{1}{\sqrt{2\pi\overline{N}(m)}} e^{-\frac{(l-\overline{N}(m))^2}{2\overline{N}(m)}}$. The uncertainty of the photon number, $\Delta N = \sqrt{\overline{N}}$, reflects the measurement uncertainty of the electron number, $\Delta N_{e,meas}$, by the propagation of uncertainty, $\Delta N_{e,meas} = \left|\frac{\partial \overline{N_e}}{\partial \overline{N}}\right| \Delta N$. Since $\frac{\partial \overline{N_e}}{\partial \overline{N}} = \frac{\overline{N_e}\hbar\omega}{\mathcal{E}_{net}}$, and using the SNR defined in Eq. (S36), the uncertainty is

$$\Delta N_{e,meas} = \frac{1}{SNR}.$$

To put this in context, consider the case of a large $\overline{N_e}$, where the electrons' Poisson distribution can be approximated as normal distribution as well. This simplifies the mathematical derivation and allows us to compare the posterior electron-number fluctuations, ΔN_e, with respect to the initial fluctuations, $\sqrt{\overline{N_e}}$.

$$\frac{1}{(\Delta N_e)^2} = \frac{1}{\overline{N_e}} + \frac{1}{\left(\Delta N_{e,meas}\right)^2},$$

Or explicitly, $\Delta N_e = \sqrt{\frac{\overline{N_e}}{1 + \overline{N_e} \cdot SNR^2}}$. Hence, the measured estimator of the photon number variance can improve the variance of the electron state to be sub-Poissonian. The measurement uncertainty-dependent electron number fluctuation is shown in Fig. S4.

S.9 Electron deflection estimation

In our proposed experimental realization, the deflection of electron depends on the amplitude of transverse component of light field (Lorentz force) and the gradient of the light field (ponderomotive force).

The amplitude of electric field can be estimate by circulating light power in resonator $P_{cir} \approx c n_{eff} \epsilon_0 A |\boldsymbol{E}|^2$, where n_{eff} is the effective refractive index of resonator, A is the cross-section area. Assume $P_{cir} = 300\text{mW}$, the racetrack resonator has a cross-section area $220\text{nm} \times 400\text{nm}$, $n_{eff} = 2$, then $|\boldsymbol{E}| \approx 2.5 \times 10^7 \text{V/m}$ and the amplitude of transverse electrical field $|E_x| \leq |\boldsymbol{E}|$.

Assuming the electron is a point particle, its quiver motion can be described classically. Let the electron moves at constant speed v_e along z-direction. Light field is given by $\boldsymbol{E}(z,t) = E_x \sin(kz - \omega t)\,\hat{\boldsymbol{x}}$, $\boldsymbol{B}(z,t) = \frac{k}{\omega} E_x \sin(kz - \omega t)\,\hat{\boldsymbol{y}}$, the phase seen by electron is $\phi(t) = kz - \omega t = \Omega t$, where $\Omega = k v_e - \omega$. The momentum changes result from Lorentz-force is $\dot{\boldsymbol{p}} = e(\boldsymbol{E} + \boldsymbol{v} \times \boldsymbol{B}) = -e E_x \frac{\Omega}{\omega} \sin(\Omega t)\,\hat{\boldsymbol{x}}$. Thus, the time-dependent momentum $\boldsymbol{p}(t) = \frac{e E_x}{\omega} \cos(\Omega t)\,\hat{\boldsymbol{x}} + \gamma m_e v_e \hat{\boldsymbol{z}}$. The deflection angle can be expressed as

$$\theta(t) \approx \frac{p_x(t)}{p_z} = \frac{eE_x}{\gamma m_e v_e \omega} \cos(\Omega t).$$

The transverse quiver motion can be expressed as

$$x(t) = \frac{eE_x}{\gamma m_e \omega \Omega} \sin(\Omega t).$$

In elastic regime (non-resonant, $\Omega \neq 0$), the maximum transverse displacement is $\frac{eE_x}{\gamma m_e \omega \Omega}$. Substituting the numerical parameters used in the main text, $v_e = 0.0063c$ (corresponding to kinetic energy 10eV), $\omega = \frac{2\pi c}{1550\text{nm}}$, yields the maximum deflection angle during interaction, $|\theta(t)| = \frac{eE_x}{\gamma m_e v_e \omega} \approx 1.9\text{mrad}$. For low speed electron, $v_e \ll c$, then $\Omega \approx -\omega$, then the maximum transverse displacement is $|x(t)| \approx \frac{eE_x}{\gamma m_e \omega^2} = 3.0\text{pm}$. Thus, the deflection can be neglected.

In the scenario of phase-mismatching, electrons do quiver motion upon the Lorentz force but will not change its kinetic energy in the end. Only ponderomotive force will change electron energy. However, the amplitude of ponderomotive force is much smaller than the amplitude of Lorentz force,

$$\left| -\frac{e^2}{4m_e\omega^2} \nabla |\boldsymbol{E}|^2 \right| \approx \frac{e^2}{4m_e\omega^2} k |\boldsymbol{E}|^2 = \frac{e^2}{4m_e c^2 k} |\boldsymbol{E}|^2 \ll e|\boldsymbol{E}|.$$

Ponderomotive Energy

$$\frac{e^2}{4m_e\omega^2} |\boldsymbol{E}|^2 = 1.9 \times 10^{-5}\text{eV}.$$

Therefore, the energy changes can also be neglected. Furthermore, one can consider resonators with a nullified-gradient waveguide to eliminate ponderomotive force, such as silicon slot waveguide.

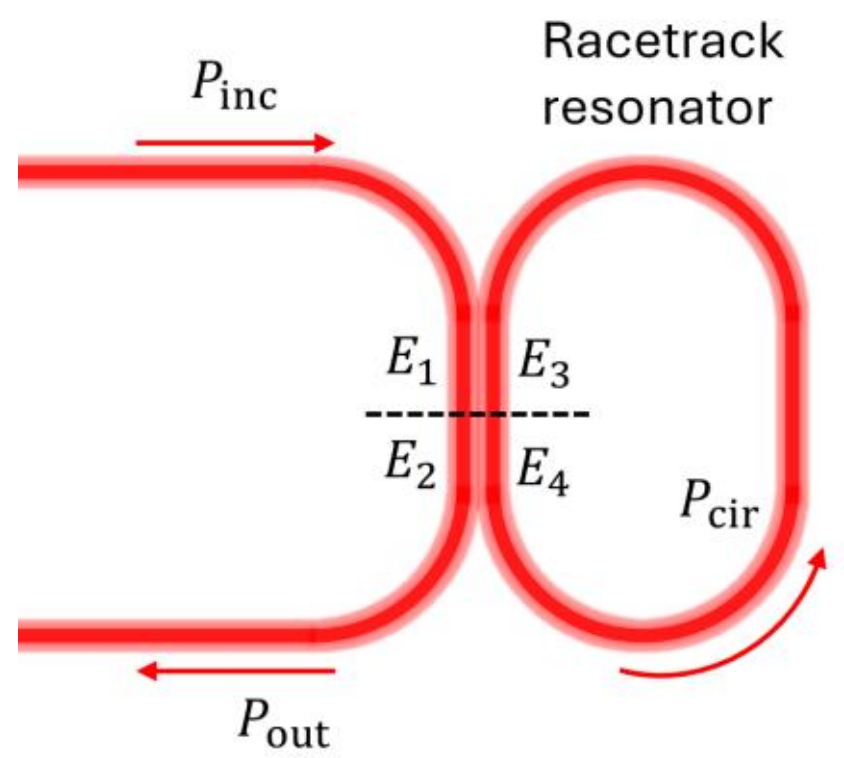

Fig. S1.
Schematically shows the incident field (E_1), the output field (E_2), and the circulating fields (E_3&E_4).

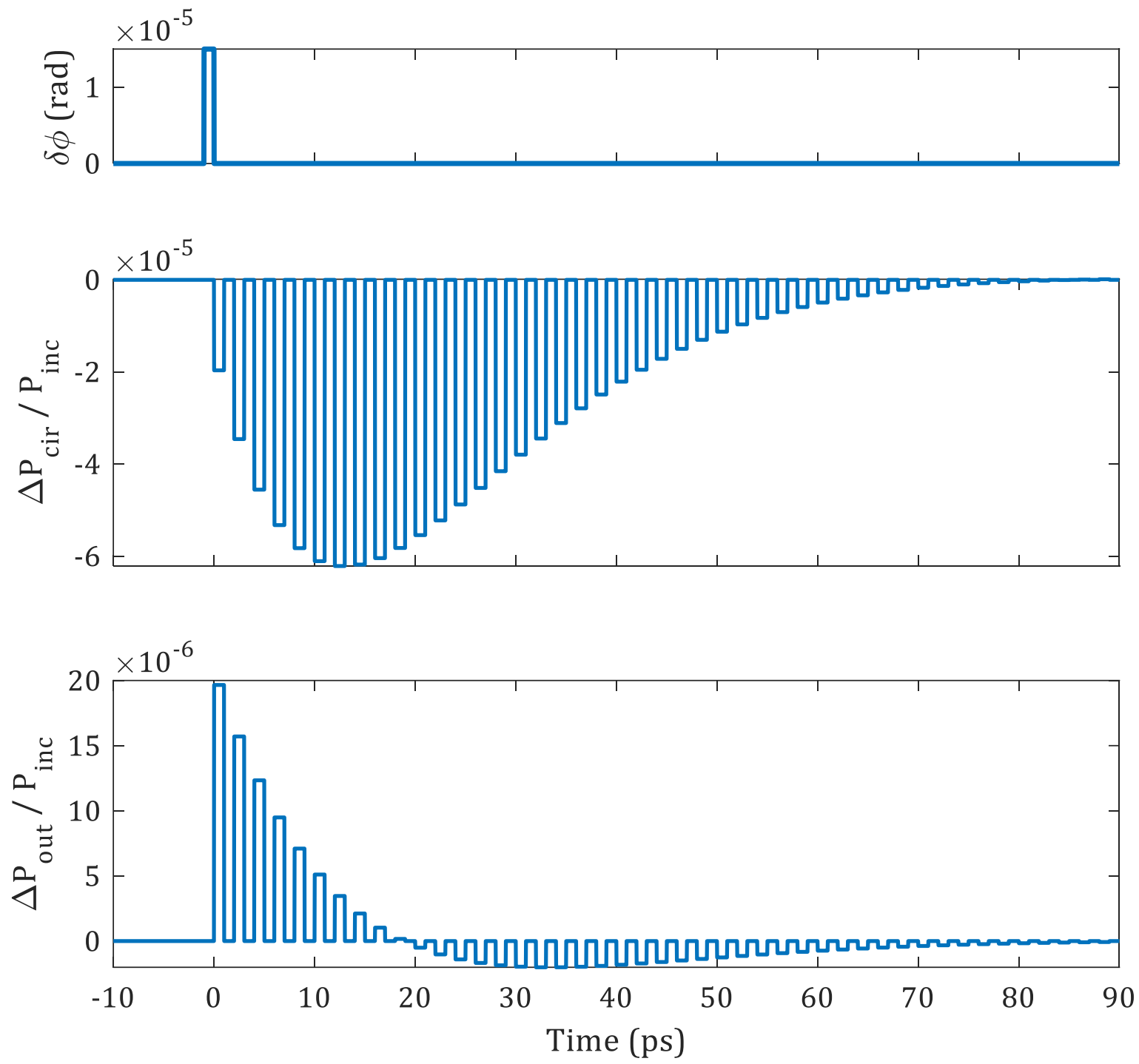

Fig. S2.

Phase shift-induced dynamics of circulating power and output power for a phase shift duration shorter than one round trip time. The width of the rectangular phase shift pulse is set to 1 ps. The resonator settings are: one round trip time is 2 ps, cavity lifetime is 50 ps, $r = 0.9$, $a = 0.9802$, finesse = 20.7.

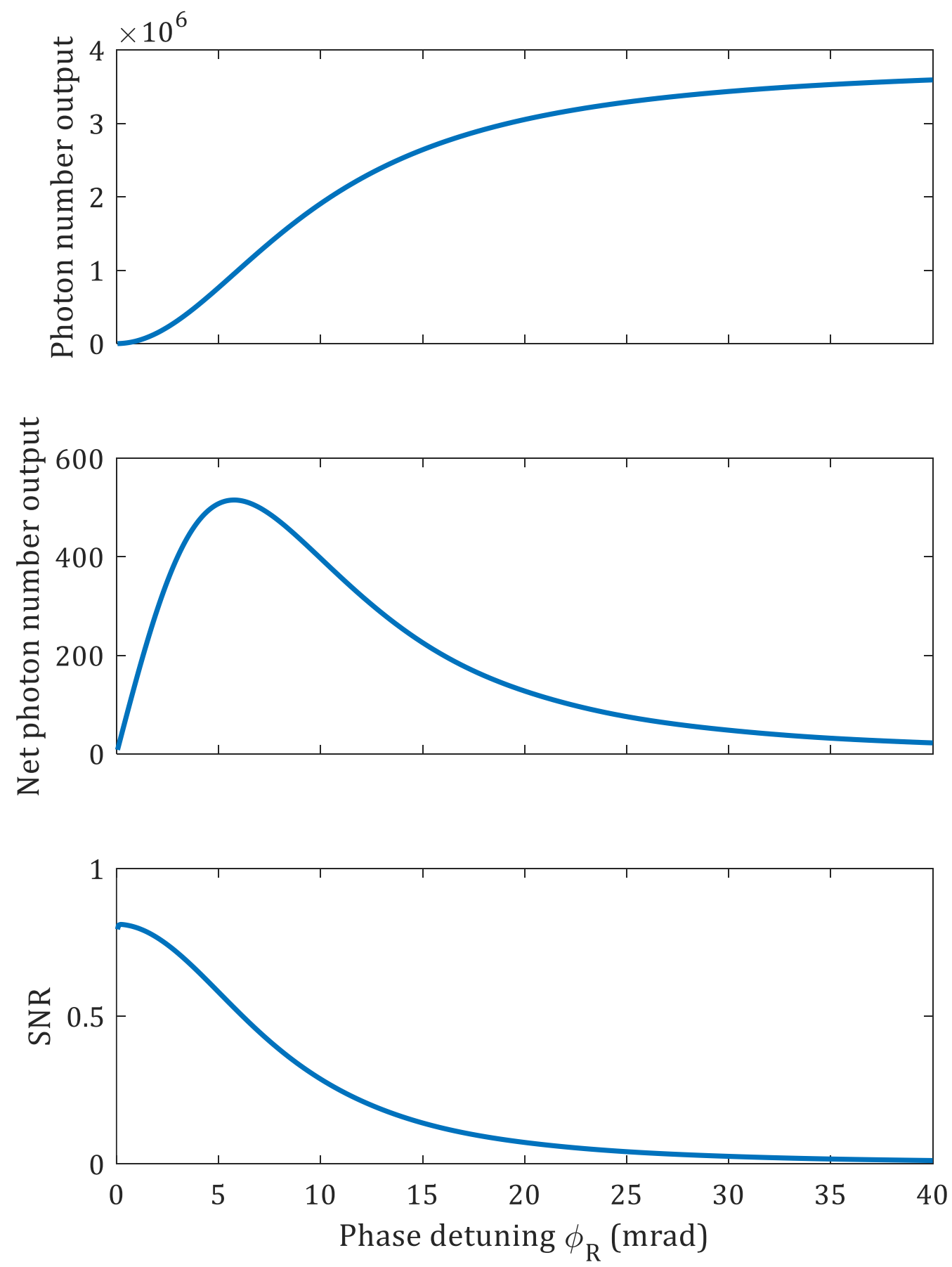

Fig. S3.

SNR analysis vs. a detuning of the roundtrip phase, ϕ_R. Total photon number output (top) at one cavity lifetime, net photon number output (middle) and SNR (bottom). The parameter settings of the resonator and $\delta\phi$ are the same as Fig. 3.

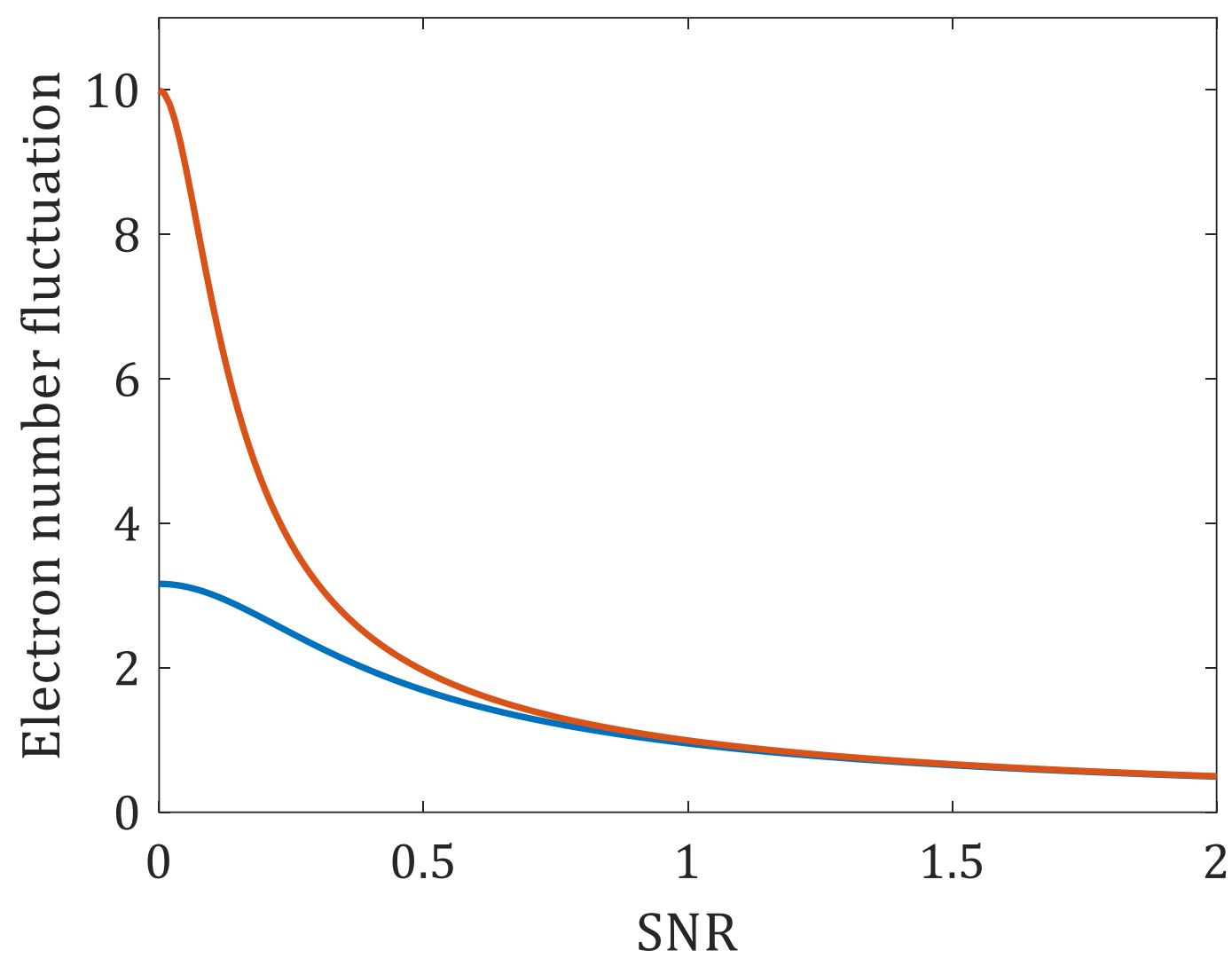

Fig. S4.
The measurement uncertainty-dependent electron number fluctuation. The initial electron number fluctuation is 10 for brown line and $\sqrt{10}$ for blue line.

REFERENCES

1. Z. Chen, Y. Jiang, Y.-T. Shao, M. E. Holtz, M. Odstrčil, M. Guizar-Sicairos, I. Hanke, S. Ganschow, D. G. Schlom, D. A. Muller, Electron ptychography achieves atomic-resolution limits set by lattice vibrations. *Science* **372**, 826–831 (2021).

2. K. Kimoto, T. Asaka, T. Nagai, M. Saito, Y. Matsui, K. Ishizuka, Element-selective imaging of atomic columns in a crystal using STEM and EELS. *Nature* **450**, 702–704 (2007).

3. B. Barwick, D. J. Flannigan, A. H. Zewail, Photon-induced near-field electron microscopy. *Nature* **462**, 902–906 (2009).

4. A. Feist, K. E. Echternkamp, J. Schauss, S. V. Yalunin, S. Schäfer, C. Ropers, Quantum coherent optical phase modulation in an ultrafast transmission electron microscope. *Nature* **521**, 200–203 (2015).

5. O. Kfir, H. Lourenço-Martins, G. Storeck, M. Sivis, T. R. Harvey, T. J. Kippenberg, A. Feist, C. Ropers, Controlling free electrons with optical whispering-gallery modes. *Nature* **582**, 46–49 (2020).

6. F. J. García De Abajo, A. Polman, C. I. Velasco, M. Kociak, L. H. G. Tizei, O. Stéphan, S. Meuret, T. Sannomiya, K. Akiba, Y. Auad, A. Feist, C. Ropers, P. Baum, J. H. Gaida, M. Sivis, H. Lourenço-Martins, L. Serafini, J. Verbeeck, A. Konečná, N. Talebi, B. M. Ferrari, C. J. R. Duncan, M. G. Bravi, I. Ostroman, G. M. Vanacore, E. Nussinson, R. Ruimy, Y. Adiv, A. Niedermayr, I. Kaminer, V. Di Giulio, O. Kfir, Z. Zhao, R. Shiloh, Y. Morimoto, M. Kozák, P. Hommelhoff, F. Barantani, F. Carbone, F. Chahshouri, W. Albrecht, S. Rey, T. Coenen, E. Kieft, H. L. Lalandec Robert, F. De Jong, M. Solà-Garcia, Roadmap for quantum nanophotonics with free electrons. *ACS Photonics* **12**, 4760–4817 (2025).

7. R. Ruimy, A. Karnieli, I. Kaminer, Free-electron quantum optics. *Nat. Phys.* **21**, 193–200 (2025).

8. F. J. García De Abajo, V. Di Giulio, Optical excitations with electron beams: Challenges and opportunities. *ACS Photonics* **8**, 945–974 (2021).

9. G. M. Vanacore, I. Madan, F. Carbone, Spatio-temporal shaping of a free-electron wave function via coherent light–electron interaction. *Riv. Nuovo Cim.* **43**, 567–597 (2020).

10. C. Roques-Carmes, S. E. Kooi, Y. Yang, N. Rivera, P. D. Keathley, J. D. Joannopoulos, S. G. Johnson, I. Kaminer, K. K. Berggren, M. Soljačić, Free-electron–light interactions in nanophotonics. *Appl. Phys. Rev.* **10**, 011303 (2023).

11. R. Dahan, S. Nehemia, M. Shentcis, O. Reinhardt, Y. Adiv, X. Shi, O. Be'er, M. H. Lynch, Y. Kurman, K. Wang, I. Kaminer, Resonant phase-matching between a light wave and a free-electron wavefunction. *Nat. Phys.* **16**, 1123–1131 (2020).

12. M. Tsarev, J. W. Thurner, P. Baum, Nonlinear-optical quantum control of free-electron matter waves. *Nat. Phys.* **19**, 1350–1354 (2023).

13. D. Zheng, S. Huang, J. Li, Y. Tian, Y. Zhang, Z. Li, H. Tian, H. Yang, J. Li, Efficiently accelerated free electrons by metallic laser accelerator. *Nat. Commun.* **14**, 5857 (2023).

14. J. H. Gaida, H. Lourenço-Martins, M. Sivis, T. Rittmann, A. Feist, F. J. García De Abajo, C. Ropers, Attosecond electron microscopy by free-electron homodyne detection. *Nat. Photonics* **18**, 509–515 (2024).

15. D. Nabben, J. Kuttruff, L. Stolz, A. Ryabov, P. Baum, Attosecond electron microscopy of sub-cycle optical dynamics. *Nature* **619**, 63–67 (2023).

16. K. E. Priebe, C. Rathje, S. V. Yalunin, T. Hohage, A. Feist, S. Schäfer, C. Ropers, Attosecond electron pulse trains and quantum state reconstruction in ultrafast transmission electron microscopy. *Nat. Photon.* **11**, 793–797 (2017).

17. G. M. Vanacore, I. Madan, G. Berruto, K. Wang, E. Pomarico, R. J. Lamb, D. McGrouther, I. Kaminer, B. Barwick, F. J. García De Abajo, F. Carbone, Attosecond coherent control of free-electron wave functions using semi-infinite light fields. *Nat. Commun.* **9**, 2694 (2018).

18. A. Feist, S. V. Yalunin, S. Schäfer, C. Ropers, High-purity free-electron momentum states prepared by three-dimensional optical phase modulation. *Phys. Rev. Res.* **2**, 043227 (2020).

19. W. Wang, D. Zheng, S. Huang, J. Li, Y. Zhang, T. Miao, S. Sun, H. Tian, H. Yang, J. Li, Energy-momentum transfer in the free-electron–photon interaction mediated by a film. *Phys. Rev. B* **109**, 134305 (2024).

20. G. M. Vanacore, G. Berruto, I. Madan, E. Pomarico, P. Biagioni, R. J. Lamb, D. McGrouther, O. Reinhardt, I. Kaminer, B. Barwick, H. Larocque, V. Grillo, E. Karimi, F. J. García De Abajo, F. Carbone, Ultrafast generation and control of an electron vortex beam via chiral plasmonic near fields. *Nat. Mater.* **18**, 573–579 (2019).

21. I. Madan, V. Leccese, A. Mazur, F. Barantani, T. LaGrange, A. Sapozhnik, P. M. Tengdin, S. Gargiulo, E. Rotunno, J.-C. Olaya, I. Kaminer, V. Grillo, F. J. G. De Abajo, F. Carbone, G. M. Vanacore, Ultrafast transverse modulation of free electrons by interaction with shaped optical fields. *ACS Photonics* **9**, 3215–3224 (2022).

22. Y. Fang, J. Kuttruff, D. Nabben, P. Baum, Structured electrons with chiral mass and charge. *Science* **385**, 183–187 (2024).

23. O. Kfir, Entanglements of electrons and cavity photons in the strong-coupling regime. *Phys. Rev. Lett.* **123**, 103602 (2019).

24. O. Kfir, V. D. Giulio, Optical coherence transfer mediated by free electrons. *Sci. Adv.* **7**, eabf6380 (2021).

25. J.-W. Henke, A. S. Raja, A. Feist, G. Huang, G. Arend, Y. Yang, F. J. Kappert, R. N. Wang, M. Möller, J. Pan, J. Liu, O. Kfir, C. Ropers, T. J. Kippenberg, Integrated photonics enables continuous-beam electron phase modulation. *Nature* **600**, 653–658 (2021).

26. V. Di Giulio, O. Kfir, C. Ropers, F. J. García De Abajo, Modulation of cathodoluminescence emission by interference with external light. *ACS Nano* **15**, 7290–7304 (2021).

27. Y. Yang, A. Massuda, C. Roques-Carmes, S. E. Kooi, T. Christensen, S. G. Johnson, J. D. Joannopoulos, O. D. Miller, I. Kaminer, M. Soljačić, Maximal spontaneous photon emission and energy loss from free electrons. *Nat. Phys.* **14**, 894–899 (2018).

28. V. Di Giulio, M. Kociak, F. J. G. De Abajo, Probing quantum optical excitations with fast electrons. *Optica* **6**, 1524 (2019).

29. Z. Zhao, Upper bound for the quantum coupling between free electrons and photons. *Phys. Rev. Lett.* **134**, 043804 (2025).

30. Z. Xie, Z. Chen, H. Li, Q. Yan, H. Chen, X. Lin, I. Kaminer, O. D. Miller, Y. Yang, Maximal quantum interaction between free electrons and photons. *Phys. Rev. Lett.* **134**, 043803 (2025).

31. V. Di Giulio, E. Akerboom, A. Polman, F. J. García De Abajo, Toward optimum coupling between free electrons and confined optical modes. *ACS Nano* **18**, 14255–14275 (2024).

32. G. Huang, N. J. Engelsen, O. Kfir, C. Ropers, T. J. Kippenberg, Electron-photon quantum state heralding using photonic integrated circuits. *PRX Quantum* **4**, 020351 (2023).

33. A. Feist, G. Huang, G. Arend, Y. Yang, J.-W. Henke, A. S. Raja, F. J. Kappert, R. N. Wang, H. Lourenço-Martins, Z. Qiu, J. Liu, O. Kfir, T. J. Kippenberg, C. Ropers, Cavity-mediated electron-photon pairs. *Science* **377**, 777–780 (2022).

34. J.-W. Henke, H. Jeng, M. Sivis, C. Ropers, Observation of quantum entanglement between free electrons and photons. arXiv:2504.13047 [quant-ph] (2025).

35. G. Arend, G. Huang, A. Feist, Y. Yang, J.-W. Henke, Z. Qiu, H. Jeng, A. S. Raja, R. Haindl, R. N. Wang, T. J. Kippenberg, C. Ropers, Electrons herald non-classical light. *Nat. Phys.* **21**, 1855–1862 (2025).

36. A. Preimesberger, S. Bogdanov, I. C. Bicket, P. Rembold, P. Haslinger, Experimental verification of electron-photon entanglement. arXiv:2504.13163 [quant-ph] (2025).

37. H. Batelaan, *Colloquium*: Illuminating the Kapitza-Dirac effect with electron matter optics. *Rev. Mod. Phys.* **79**, 929–941 (2007).

38. D. L. Freimund, K. Aflatooni, H. Batelaan, Observation of the Kapitza–Dirac effect. *Nature* **413**, 142–143 (2001).

39. P. L. Kapitza, P. A. M. Dirac, The reflection of electrons from standing light waves. *Math. Proc. Camb. Phil. Soc.* **29**, 297–300 (1933).

40. O. Schwartz, J. J. Axelrod, S. L. Campbell, C. Turnbaugh, R. M. Glaeser, H. Müller, Laser phase plate for transmission electron microscopy. *Nat. Methods* **16**, 1016–1020 (2019).

41. O. Schwartz, J. J. Axelrod, D. R. Tuthill, P. Haslinger, C. Ophus, R. M. Glaeser, H. Müller, Near-concentric Fabry-Pérot cavity for continuous-wave laser control of electron waves. *Opt. Express* **25**, 14453–14462 (2017).

42. H. Müller, J. Jin, R. Danev, J. Spence, H. Padmore, R. M. Glaeser, Design of an electron microscope phase plate using a focused continuous-wave laser. *New J. Phys.* **12**, 073011 (2010).

43. J. J. Axelrod, S. L. Campbell, O. Schwartz, C. Turnbaugh, R. M. Glaeser, H. Müller, Observation of the relativistic reversal of the ponderomotive potential. *Phys. Rev. Lett.* **124**, 174801 (2020).

44. F. J. García De Abajo, A. Konečná, Optical modulation of electron beams in free space. *Phys. Rev. Lett.* **126**, 123901 (2021).

45. M. C. Chirita Mihaila, P. Weber, M. Schneller, L. Grandits, S. Nimmrichter, T. Juffmann, Transverse electron-beam shaping with light. *Phys. Rev.* **12**, 031043 (2022).

46. M. C. C. Mihaila, N. Laštovičková Streshkova, M. Kozák, Light-based chromatic aberration correction of ultrafast electron microscopes. *Phys. Rev. Lett.* **134**, 203802 (2025).

47. M. C. Chirita Mihaila, P. Koutenský, K. Moriová, M. Kozák, Light-based electron aberration corrector. *Nat. Photonics*, doi: (2025), **19**, 1309, 1314.

48. Z. Nekula, T. Juffmann, A. Konečná, Compensating spherical and chromatic aberrations of ultrafast electron microscopes with laser beams. *Phys. Rev. Res.* **7**, 043024 (2025).

49. M. O. Scully, M. S. Zubairy, *Quantum Optics* (Cambridge Univ. Press, ed. 1, 1997).

50. P. Grangier, J. A. Levenson, J.-P. Poizat, Quantum non-demolition measurements in optics. *Nature* **396**, 537–542 (1998).

51. M. Brune, S. Haroche, V. Lefevre, J. M. Raimond, N. Zagury, Quantum nondemolition measurement of small photon numbers by Rydberg-atom phase-sensitive detection. *Phys. Rev. Lett.* **65**, 976–979 (1990).

52. C. Guerlin, J. Bernu, S. Deléglise, C. Sayrin, S. Gleyzes, S. Kuhr, M. Brune, J.-M. Raimond, S. Haroche, Progressive field-state collapse and quantum non-demolition photon counting. *Nature* **448**, 889–893 (2007).

53. V. Di Giulio, F. J. García De Abajo, Optical-cavity mode squeezing by free electrons. *Nanophotonics* **11**, 4659–4670 (2022).

54. V. Di Giulio, F. J. G. De Abajo, Electron diffraction by vacuum fluctuations. *New J. Phys.* **22**, 103057 (2020).

55. J. Zak, Berry's phase for energy bands in solids. *Phys. Rev. Lett.* **62**, 2747–2750 (1989).

56. M. V. Berry, Quantal phase factors accompanying adiabatic changes. *Proc. R. Soc. Lond. A Math. Phys. Sci.* **392**, 45–57 (1984).

57. V. B. Braginsky, Y. I. Vorontsov, K. S. Thorne, Quantum nondemolition measurements. *Science* **209**, 547–557 (1980).

58. M. Kitagawa, N. Imoto, Y. Yamamoto, Realization of number-phase minimum-uncertainty states and number states by quantum nondemolition measurement. *Phys. Rev. A* **35**, 5270–5273 (1987).

59. V. B. Braginsky, F. Ya. Khalili, Quantum nondemolition measurements: The route from toys to tools. *Rev. Mod. Phys.* **68**, 1–11 (1996).

60. G. Breitenbach, S. Schiller, J. Mlynek, Measurement of the quantum states of squeezed light. *Nature* **387**, 471–475 (1997).

61. D. F. Walls, Squeezed states of light. *Nature* **306**, 141–146 (1983).

62. A. Ourjoumtsev, H. Jeong, R. Tualle-Brouri, P. Grangier, Generation of optical 'Schrödinger cats' from photon number states. *Nature* **448**, 784–786 (2007).

63. M. Lewenstein, M. F. Ciappina, E. Pisanty, J. Rivera-Dean, P. Stammer, Th. Lamprou, P. Tzallas, Generation of optical Schrödinger cat states in intense laser–matter interactions. *Nat. Phys.* **17**, 1104–1108 (2021).

64. Y.-R. Chen, H.-Y. Hsieh, J. Ning, H.-C. Wu, H. L. Chen, Z.-H. Shi, P. Yang, O. Steuernagel, C.-M. Wu, R.-K. Lee, Generation of heralded optical cat states by photon addition. *Phys. Rev. A* **110**, 023703 (2024).

65. J. P. Dowling, Quantum optical metrology—The lowdown on high-N00N states. *Contemp. Phys.* **49**, 125–143 (2008).

66. I. Afek, O. Ambar, Y. Silberberg, High-NOON states by mixing quantum and classical light. *Science* **328**, 879–881 (2010).

67. T. W. B. Kibble, Mutual refraction of electrons and photons. *Phys. Rev.* **150**, 1060–1069 (1966).

68. T. W. B. Kibble, Refraction of electron beams by intense electromagnetic waves. *Phys. Rev. Lett.* **16**, 1054–1056 (1966).

69. K. J. Vahala, Optical microcavities. *Nature* **424**, 839–846 (2003).

70. Z. Yao, K. Wu, B. X. Tan, J. Wang, Y. Li, Y. Zhang, A. W. Poon, Integrated silicon photonic microresonators: Emerging technologies. *IEEE J. Sel. Top. Quantum Electron.*, **6**, 5900324 (2018).

71. L. Cai, J. Pan, Y. Zhao, J. Wang, S. Xiao, Whispering gallery mode optical microresonators: Structures and sensing applications. *Phys. Status Solidi A* **217**, 1900825 (2020).

72. J. Nijem, A. Naiman, R. Zektzer, C. Frydendahl, N. Mazurski, U. Levy, High-Q and high finesse silicon microring resonator. *Opt. Express* **32**, 7896–7906 (2024).

73. L. Zhang, L. Jie, M. Zhang, Y. Wang, Y. Xie, Y. Shi, D. Dai, Ultrahigh-Q silicon racetrack resonators. *Photon. Res.* **8**, 684 (2020).

74. J. Heebner, R. Grover, T. Ibrahim, *Optical Microresonators* (Springer New York, 2008), vol. 138.

75. V. Giovannetti, S. Lloyd, L. Maccone, Quantum-enhanced measurements: Beating the standard quantum limit. *Science* **306**, 1330–1336 (2004).

76. S. Even-Haim, E. Nussinson, R. Ben-Maimon, A. Gorlach, R. Ruimy, E. Shahmoon, O. Schwartz, I. Kaminer, Spin squeezing in electron microscopy. arXiv:2507.09243 [quant-ph] (2025).

77. C. I. Velasco, F. J. García De Abajo, Free-space optical modulation of free electrons in the continuous-wave regime. *Phys. Rev. Lett.* **134**, 123804 (2025).

78. L. Dupays, J.-C. Pain, Closed forms of the Zassenhaus formula. *J. Phys. A Math. Theor*. **56**, 255202 (2023).

79. W. Magnus, On the exponential solution of differential equations for a linear operator. *Commun. Pure Appl. Math.* **7**, 649–673 (1954).

80. E. Cohen, H. Larocque, F. Bouchard, F. Nejadsattari, Y. Gefen, E. Karimi, Geometric phase from Aharonov–Bohm to Pancharatnam–Berry and beyond. *Nat. Rev. Phys.* **1**, 437–449 (2019).
